# Integrated genomic analysis reveals regulatory pathways and dynamic landscapes of the tRNA transcriptome

**DOI:** 10.1038/s41598-021-83469-6

**Published:** 2021-03-04

**Authors:** Zefang Sun, Jia Tan, Minqiong Zhao, Qiyao Peng, Mingqing Zhou, Shanru Zuo, Feilong Wu, Xueguang Li, Yangyang Dong, Ming Xie, Yide Yang, Junhua Zhou, Xianghua Liu, Quanze He, Zuping He, Xing Yu, Quanyuan He

**Affiliations:** 1 The Key Laboratory of Model Animals and Stem Cell Biology in Hunan Province, School of Medicine, Hunan Normal University, Tongzipo Road 371, Changsha, 410013 Hunan People’s Republic of China; 2 Verna and Marrs McLean Department of Biochemistry and Molecular Biology, Baylor College of Medicine One Baylor Plaza, Houston, TX 77-30 USA; 3 The Affiliated Suzhou Hospital of Nanjing Medical University, Suzhou, People’s Republic of China

**Keywords:** Cancer genomics, Gene regulation, Gene regulatory networks

## Abstract

tRNAs and tRNA-derived RNA fragments (tRFs) play various roles in many cellular processes outside of protein synthesis. However, comprehensive investigations of tRNA/tRF regulation are rare. In this study, we used new algorithms to extensively analyze the publicly available data from 1332 ChIP-Seq and 42 small-RNA-Seq experiments in human cell lines and tissues to investigate the transcriptional and posttranscriptional regulatory mechanisms of tRNAs. We found that histone acetylation, cAMP, and pluripotency pathways play important roles in the regulation of the tRNA gene transcription in a cell-specific manner. Analysis of RNA-Seq data identified 950 high-confidence tRFs, and the results suggested that tRNA pools are dramatically distinct across the samples in terms of expression profiles and tRF composition. The mismatch analysis identified new potential modification sites and specific modification patterns in tRNA families. The results also show that RNA library preparation technologies have a considerable impact on tRNA profiling and need to be optimized in the future.

## Introduction

tRNAs are well known as adaptor molecules that transport amino acids to the ribosomes for protein synthesis and are considered archetypal housekeeping molecules. Recent evidence suggests that tRNAs perform additional functions, such as acting as signaling molecules in numerous metabolic and cellular processes in both prokaryotes and eukaryotes, and are implicated in the translational regulation of mRNA expression, animal development, and diseases^1–3^. Intriguingly, a class of small ncRNAs derived from mature tRNAs and their precursors, known as tRNA-derived fragments (tRFs), was discovered by recent deep sequencing and recognized as a major RNA species in human cells^4, 5^. Increasing evidence suggests that tRFs are not byproducts of random degradation but rather are functional molecules that regulate translation and gene expression^4, 6, 7^ related to development^8^ and human diseases^9–11^.

Numerous studies have explored tRNA biology; however, the global mechanism of regulation of tRNA genes and their transcripts is unclear^11^. Some fundamental questions to be elucidated are as follows. (1) What are the major mechanisms regulating tRNA transcription? (2) What are the differences in tRNA/tRF profiles across various cells and tissues? (3) What are the functions of tRFs?

Next-generation ChIP-Seq and RNA-Seq technologies are powerful tools used to decode the mechanisms of tRNA regulation and expression profiles in the cells. However, tRNAs have extensive posttranscriptional modifications and secondary structures; thus, use of the regular NGS technology to explore tRNA profiles remains a major challenge. Recently, several improved experimental and bioinformatics methods for small RNA sequencing (tRNA-Seq) have been introduced to overcome certain technical limitations^12–15^. For example, demethylation of the bases of tRNAs (DM-tRNA-Seq^14^, ARM-Seq^15^) and tRNA fragments (Hydro-tRNAseq^16^) were used to reduce sequencing bias resulting from posttranscriptional modifications or secondary structure. The Iso-tRNA-CP algorithm was designed to evaluate the relative expression levels of the tRNA genes based on their proportional transcript contribution to a corresponding isodecoder set to reduce the effects of sequencing bias^17^.

In this study, we analyzed publicly available large-scale NGS data from 1332 ChIP-Seq, 8 DM-tRNA-Seq, and 34 small-RNA Seq experiments to explore the mechanisms of transcriptional regulation of the tRNA genes and tRNA/tRF profiles in human cell lines and tissues (Fig. S1). More than 68 transcription factors and chromatin remodelers enriched in tRNA genes were identified in three human cell lines. The data show that tRNA transcription is tightly regulated by several disease-related pathways. Histone modifications, especially histone acetylation, may play important roles in the regulation of tRNA transcription. We used a new tRNAExplorer algorithm to identify 950 high-confidence tRFs in human cell lines and tissues. Comparison of tRNA profiles across the samples suggests that the profiles are dramatically distinct from each other in terms of expression and tRF composition. Certain new 5′-additions of tRFs, such as T_−1_-addition, and 8 new potential modification sites were identified. Additionally, we found that tRNA cleavage sites are very conserved across the samples and clustered on exposed surfaces of tRNAs.

## Materials and methods

### Data collection and preprocessing

The human hg38 genome sequence (FASTA) and annotation (GTF) files were downloaded from the UCSC ftp server (https://genome.ucsc.edu/goldenPath). The definitions of the tRNA genes based on the GtRNAdb database^13^ were downloaded from the UCSC table browser (https://genome.ucsc.edu/cgi-bin/hgTables). Structural information on the tRNA genes was predicted by tRNAScan-SE (http://lowelab.ucsc.edu/tRNAscan-SE/)^13^. Mitochondrial tRNA genes, pseudo tRNA genes, and genes with tRNAScan scores less than 30 were removed. The tRNA genes/transcripts were grouped into six levels based on the amino acids they carry, anticodons and sequence (Table S1).

ChIP-Seq data (bigWig files) were downloaded from the GEO database (https://www.ncbi.nlm.nih.gov/geo/) (93 for h1-ESCs, 527 for HepG2 cells, and 712 for K562 cells; the details can be found in Supplemental Data Files S10–S12)^18^. All Chip-Seq datasets had at least 20 M reads with read lengths over 37 bp.

In this study, we analyzed two RNA-Seq datasets: DM-tRNA-Seq and Cell Lines-Tissues-Seq datasets. The DM-tRNA-Seq dataset was downloaded from the ENA database (https://www.ebi.ac.uk/ena/browser/view/PRJNA277309) and contained four samples with two technical repeats of purified tRNA and total RNA as templates with (+) or without (−) demethylase treatment (total RNA control, total RNA treatment, tRNA control, and tRNA treatment)^14^. The Cell Lines-Tissues dataset was downloaded from the ENCODE database (https://www.encodeproject.org) and included 48 small-RNA-Seq datasets from 4 human cell lines and 8 tissues (Supplemental Data File S9). To minimize the batch effect, all small-RNA sequencing experiments selected were performed with the same cDNA library construction methods (https://www.encodeproject.org/experiments/ENCSR000CRF/) and met three criteria: (1) the purified RNAs were size-selected to be shorter than 200 bp; (2) the read length was at least 100 base pairs; and (3) each FASTA file had 30 million aligned reads. Notably, the cDNA libraries of the DM-tRNA-Seq and Cell Lines-Tissues datasets were generated by template switching reaction and regular A-tailing cDNA synthesis method, respectively, which could have significantly influenced the results of tRNA profiling (see below).

### ChIP-Seq data analysis

All ChIP-Seq data (bigWig files) available for each gene were combined for each cell line to achieve higher signal/noise ratios. Then, the combined bigWig files were normalized to the total number of mapped reads in the FASTQ file. To calculate the binding intensities for the matrix, we summed the pileup area around the transcriptional start site (TSS) of the tRNA genes within 100 bp up/downstream using the computeMatrix function in deepTools2^19^ under a reference-point model. The matrix and binding profiles were generated by R and deepTools2^19^ (Supplemental Data Files S14 and S15).

### Definitions and classification of tRNA and tRFs

GtRNAdb gene symbols were used to identify the tRNA genes (http://gtrnadb.ucsc.edu/docs/naming/). The definitions of the tRNA hierarchy (gene- > isodecoders- > isoacceptors) were adapted from previous studies (Table S1)^20, 21^. All tRNA genes sharing the same mature sequence were grouped into a tRNA family. The IDs of the tRNA families followed the pattern “tRFM#tRNA_ID” (e.g., tRFM#tRNA-Glu-CTC-1-1), in which “tRFM” is the ID prefix of the tRNA families and tRNA_ID is the GtRNAdb gene symbol of a member that has the smallest transcript ID and gene locus ID. If a tRNA does not share its mature sequence with any other tRNA, it was assigned to a family containing only itself.

In this study, we defined tRFs as any RNA fragments that are cleavage products of the transcripts of the mitochondrial and nuclear tRNA genes. Therefore, full-length mature tRNAs were also considered a type of tRF. Structurally, tRFs fall into 12 distinct classes based on their overlapping range in the tRNA genes within up- and downstream sequences (60 bp UTRs) (Fig. 3A). Specifically, Full_tRNA and Full_U_tRNA represent full-length tRNAs and their precursors. The character “U” indicates that these tRFs contain the UTR sequences. The 5_tRNA_halves, 5_U_tRNA_halves, 3_tRNA_halves, and 3_U_tRNA_halves species correspond to four classes of the products of cleavage within the anticodon of mature tRNAs or their precursors. The 5_U_tRF and 3_U_tRF species correspond to the fragments that overlap with the upstream or downstream regions (UTRs) of mature tRNA but are shorter than 5_U_tRNA_halves and 3_U_tRNA_halves. The 5_tRFs and 3_tRFs species are shorter than 5_tRNA_halves and 3_tRNA_halves, respectively. Internal tRFs (i_tRFs) are remaining fragments derived from mature tRNAs. Finally, the “other” class represents all remaining tRFs, for example, tRFs that are only mapped upstream or downstream of the tRNA genes. The default minimum length of tRFs was set as 18 nts. The IDs of tRFs are composed of the pattern “tRF#Len-SeqCODE” (e.g., tRF#23-ZKXU53K80E), where ‘tRF’ is a prefix for the ID type, ‘Len’ (e.g., 23) indicates the length of tRFs, and ‘SeqCODE’ (e.g., ZKXU53K80E) is the sequence zip code generated by the Python code from MINTbase^22^.

### tRF sequencing analysis

Current tRNA-Seq bioinformatics tools have many limitations^12, 23^; thus, we developed a new program, tRNAExplorer, to process the tRNA-Seq data. Initially, low-quality reads were filtered out, and adapters were trimmed by Trimmomatic^24^. Then, BLASTN was used to search the reads against a customized tRNA transcript database that contained four major types of tRNAs to maximize the mapping possibility: (1) tRNA precursors with intron(s), (2) tRNA precursors without intron(s), (3) mature tRNAs, and (4) mature tRNAs with CCA. Only the best hits with identity over 96% were kept for subsequent analyses. The multiple aligned reads were equally assigned to related tRNAs. All tRFs were categorized into 12 classes based on the rules mentioned in the previous paragraph. The minimum requirements for tRF identification included the following criteria: (1) minimum identity of 96% between a tRF and its database sequence match, (2) longer than 18 nt, and (3) supported by more than 500 reads in at least one sample. High-confidence tRFs should be supported by more than 1000 reads in at least two samples. The relative abundance of a tRNA is represented by the normalized read number (NR), which is calculated as follows: NR = M × 10^9^/N, where M indicates the number of tRNA mapping reads and N indicates total reads in the FASTQ file. The program can generate analysis reports (several tsv files) for the expression matrix mismatch and cleavage site statistics of tRNA/tRFs across the samples. This information can be used to analyze the modifications and terminal addition patterns of tRNAs. Finally, tRNAExplorer also presents a Python kernel to implement more than 11 regular analyses. Most of plots in the manuscript including sample correlation, clustered matrix of tRNA/tRFs, tRF classification, pileup profiles of tRNAs, and others were prepared by the kernel. (https://github.com/hqyone/tRNAExplorer/blob/master/help/tRNAExplorer_manual.md). The results of the comparison between tRNAExplorer and current tools (such as MINTmap and tDRMapper) suggest its superiority in terms of the sensitivity of tRF identification, analysis, and running speed (data not shown). The source code and documentation for tRNAExplorer can be downloaded at the GitHub site (https://github.com/hqyone/tRNAExplorer).

### Protein interaction network analysis

Protein–protein interactions were analyzed using the STRING server (https://string-db.org/)^25^. Functional annotations of the genes were downloaded from the UniProt database (https://www.uniprot.org)^26^.

## Results

### Transcriptional regulatory matrixes of tRNA genes

**Figure 1:**
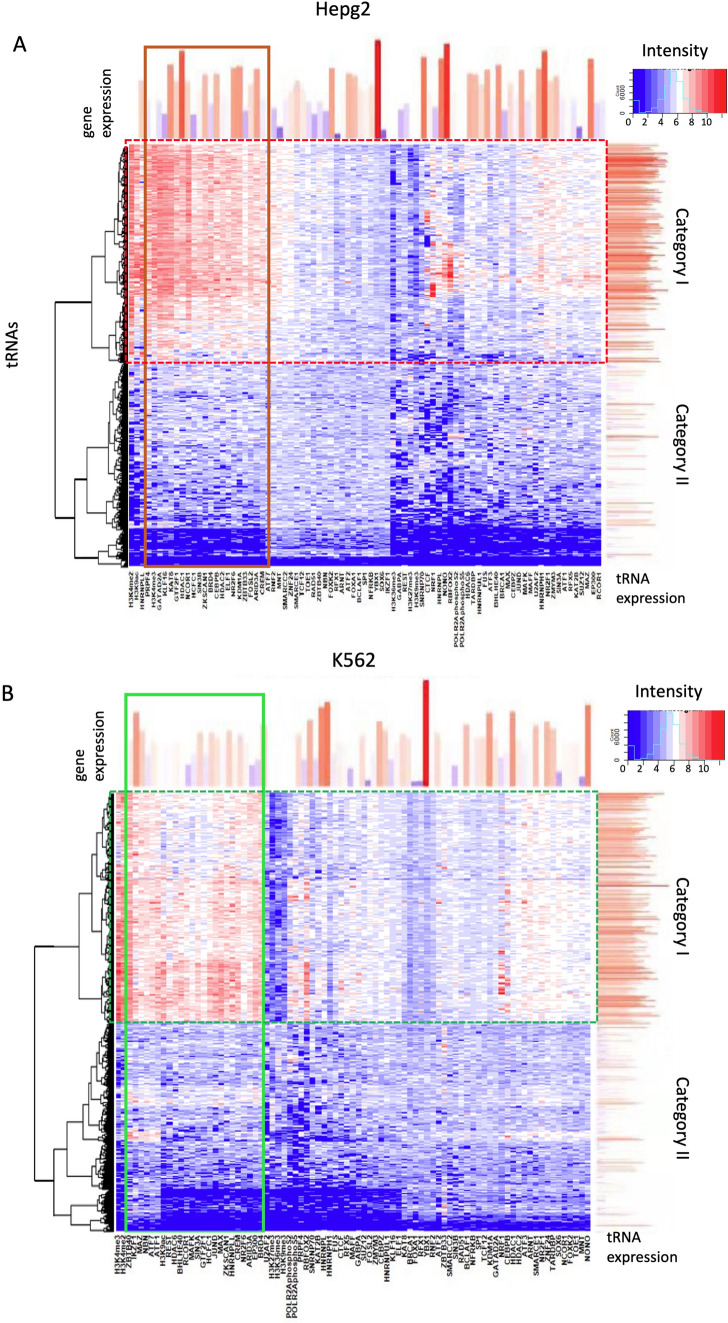
ChIP-Seq matrixes of tRNA genes in HepG2 and K562 cells. (**A**, **B**) ChIP-Seq profiles of 83 DBPs and six histone modifications of tRNA genes in HepG2 and K562 cells, respectively. The red blocks in the matrices indicate higher binding intensities of DBPs or enrichment of histone markers. The red bar charts at the top and right side of the matrixes represent the abundance of DBPs and tRNAs, respectively. The solid red and green rectangles indicate the top tRNA gene-binding proteins in HepG2 and K562 cell lines, and the dotted red and green rectangles highlight category I tRNAs in the two cell lines.

tRNA transcription is known to be driven by the Pol-III system; however, little is known about the control mechanism of tRNA transcription. To partially answer this question, we analyzed more than 1332 ChIP-seq data from three cell lines (K562, HepG2, and H1-ESC) to identify the tRNA gene-binding factors and related epigenetic markers. We calculated the binding/enrichment intensities of these factors and markers on the tRNA genes by directly normalizing the number of reads aligned to the corresponding promoters to the total aligned read count in the samples. The normalized read counts were used to construct the hierarchical clustering matrixes of 625 tRNAs across 253 (K562), 264 (HepG2), and 89 (H1-ESC) DBPs or histone markers (Figs. S2–S4 and “Supplemental Data”). Based on these results, we identified 85, 76 and 25 proteins bound to the tRNA genes in HepG2, K562, and H1-ES cells, respectively (see Tables S2–Table S4). We also constructed and compared two matrices, including 625 tRNAs across 89 factors shared by K562 and HepG2 cells (Fig. 1A,B, Tables S2, Table S3).

**Figure 2:**
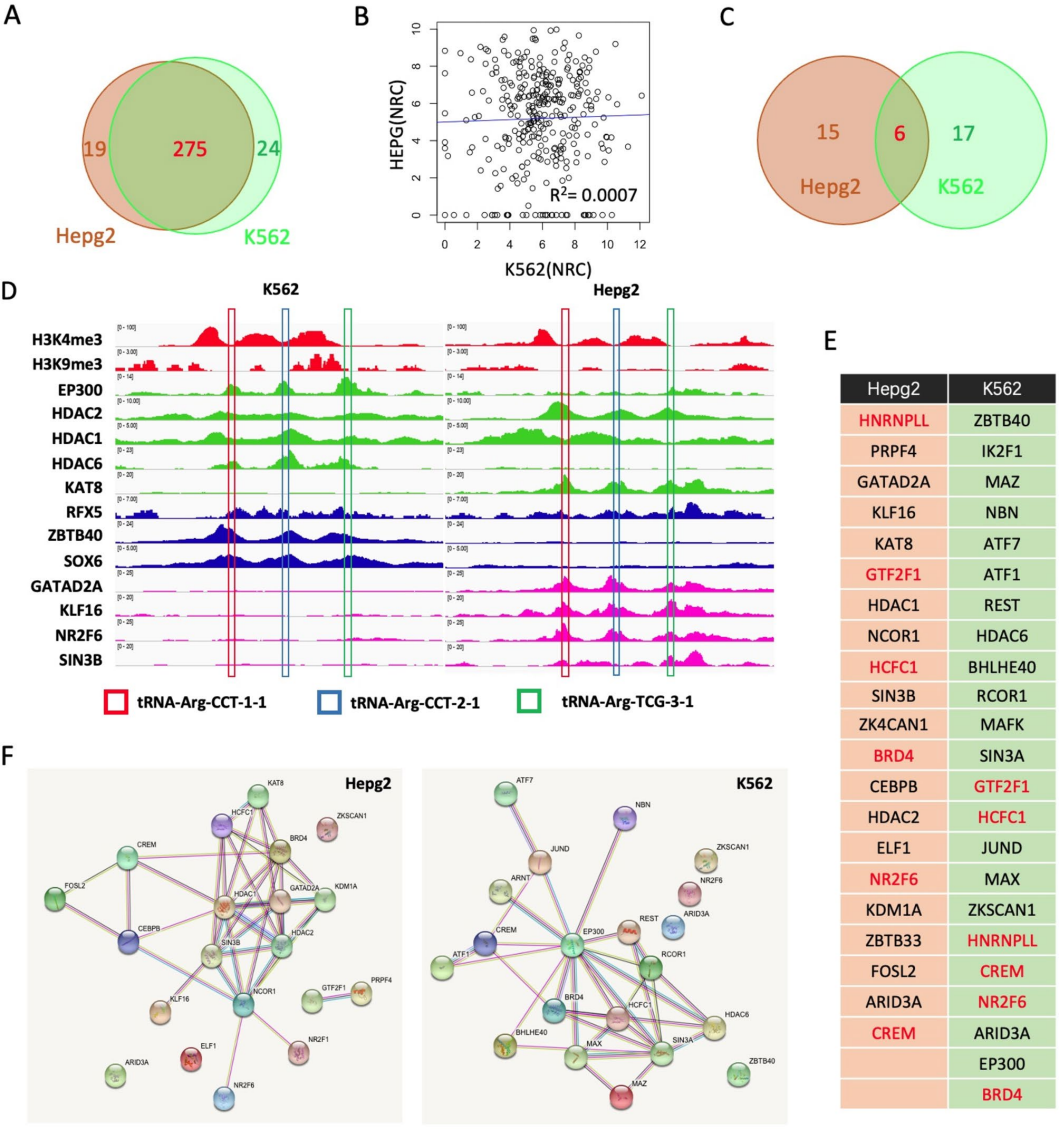
Comparison of tRNA regulation networks of K562 and HepG2 cells. (**A**) Venn diagram showing the overlapping category I tRNAs in two cell lines. (**B**) Venn diagram of the top tRNA-binding DBPs in two cell lines. (**C**) Correlation of the expression level (normalized read counts) of category I tRNAs in two cell lines. Each dot represents a tRNA gene, and R-squared indicates to what extent the variance in K562 cells explains the variance in HepG2 cells. (**D**) A snapshot of aligned ChIP-Seq profiles of two histone methylation markers and 12 DBPs of three tRNA genes in two cell lines. The red, blue, and green rectangles indicate the locations of tRNA-Arg-CCT-1-1, tRNA-Arg-CCT-2-1, and tRNA-Arg-CCG-3-1, respectively, which are very close to each other. (**E**) A table listing the top tRNA gene-binding DBPs in two cell lines; shared DBPs are red. (**F**) The protein–protein interaction/association networks for top DBPs in HepG2 and K562 cells constructed by the STRING server (https://string-db.org/) with default parameters.

Analysis of the matrices indicated that tRNA genes can be clearly divided into two categories based on their binding/enrichment profiles. In category I, the tRNA genes (294 in HepG2 and 299 in K562 cell lines) bind more than 20 DNA-binding proteins (DBPs). Another category includes tRNA genes with low or even absent binding signals for all target proteins. Combination with RNA-Seq data clearly indicated that the tRNA genes of the first category have significantly higher expression levels on average than that of the tRNA genes of the second category. Although most category 1 tRNA genes (275) were shared by the two cell lines (Fig. 2A), there was no correlation between their expression levels in the two cell lines (Fig. 2B). The top tRNA gene-binding factors (sorted by relative binding intensity) were also dramatically different between the categories (Fig. 2C,D). These results suggest that distinct DBP binding profiles are major contributors to the cellular specificity of tRNA profiles. The heatmaps and snapshots of aligned ChIP-Seq profiles of differential histone epigenetic markers and DBPs clearly show the details of distinct DBP binding profiles in the K562 and HepG2 cell lines (Fig. 2E and Fig. S5).

Subsequent investigation of the cellular functions of the top tRNA gene-binding factors surprisingly indicated that both transcriptional activators and repressors cobind to the active tRNA genes (category I tRNA genes) in the two cell lines (Fig. 2D). For example, two key components of the SIN3A repression complex (SIN3A and RCOR1) and two transcriptional activators (ATF7 and ATF1) cobind to the tRNA genes in K562 cells. These phenomena were also observed in the case of histone acetylation enzymes (see below).

### Histone modifications regulate tRNA transcription

Investigation of the ChIP-Seq profiles of histone modifications indicated that active markers (such as H3K4m3 and H3K9ac) were highly enriched and transcriptional repression markers (such as H3K9me3, H3K27me3, and H3K30me3) were absent at the promoters of category I tRNA genes (Fig. S5). Additionally, there were no significant differences between the two cell lines in terms of H3K4me3 and H3K9me3 profiles (Fig. S5A), suggesting that the modifications may function as basic mechanisms to enable the transcriptional potential of the tRNA genes as they did in regular genes.

Furthermore, the data showed that both histone acetyltransferase (EP300, KAT8) and histone deacetylases (HDAC1, HDAC2, and HDAC6) bind to the tRNA genes in combination with associated proteins (BRD4 for EP300 and REST, SIN3A, SIN3B, and RCOR1 for HDACs). For example, EP300 binds to the tRNA genes with HDAC1 and HDAC6 in K562 cells (Fig. 2D). KAT8 and HDAC2 cobind to the same tRNA genes in HepG2 cells. Surprisingly, the binding intensity of some subunits of HDAC complexes (such as HDAC6 and SIN3B) showed a relatively high positive correlation with tRNA gene expression in K562 and HepG2 cells (Fig. S5E,F). In H1-ES cells, ATF2, a histone acetyltransferase, binds to the tRNA genes in combination with HDAC6 (Fig. S4). Furthermore, the interaction network analyses clearly indicated that the EP300-SIN3A-centered and HDAC1/2-KAT6-centered networks are the major tRNA regulatory components in K562 and HepG2 cells, respectively (Fig. 2F). This finding supports a competition model for both sites and suggests that histone acetylation may play an important role in the regulation of tRNA transcription.

### Other signaling pathways regulating tRNA transcription

In addition to histone acetylation, our data highlighted other signaling pathways that may be involved in tRNA regulation. For example, ATF1 and ATF7, two key factors of the cAMP signaling pathway^27, 28^, were found associating with the tRNA genes in K562 cells. This observation raise the possibility that the tRNA pathway can be controlled by the cAMP pathway. Additionally, both MAX and MAZ can bind to the tRNA genes in K562 cells. These factors are the key components of the MLL complex and form transcriptionally activated complexes with the proto-oncogene Myc to contribute to autonomous proliferation and growth. High expression of tRNAs has been linked to cell transformation and proliferation^10, 29, 30^; thus, it is of interest to test whether the cMyc or MLL pathways can promote tRNA transcription in cancer cells. Additionally, in H1 embryonic stem cells, POU5F1 (OCT4) and Nanog, two key pluripotency transcription factors, were bound to the tRNA genes, suggesting that the tRNA pathway may be a key component of the pluripotency network^31, 32^ (Fig. S4). Overall, our data suggest that the tRNA pathway is regulated by a substantially higher number of regulatory mechanisms than assumed previously (Fig. S5, Table S4) in a cell-specific manner.

### tRNA profiles revealed by DM-tRNA-Seq

**Figure 3:**
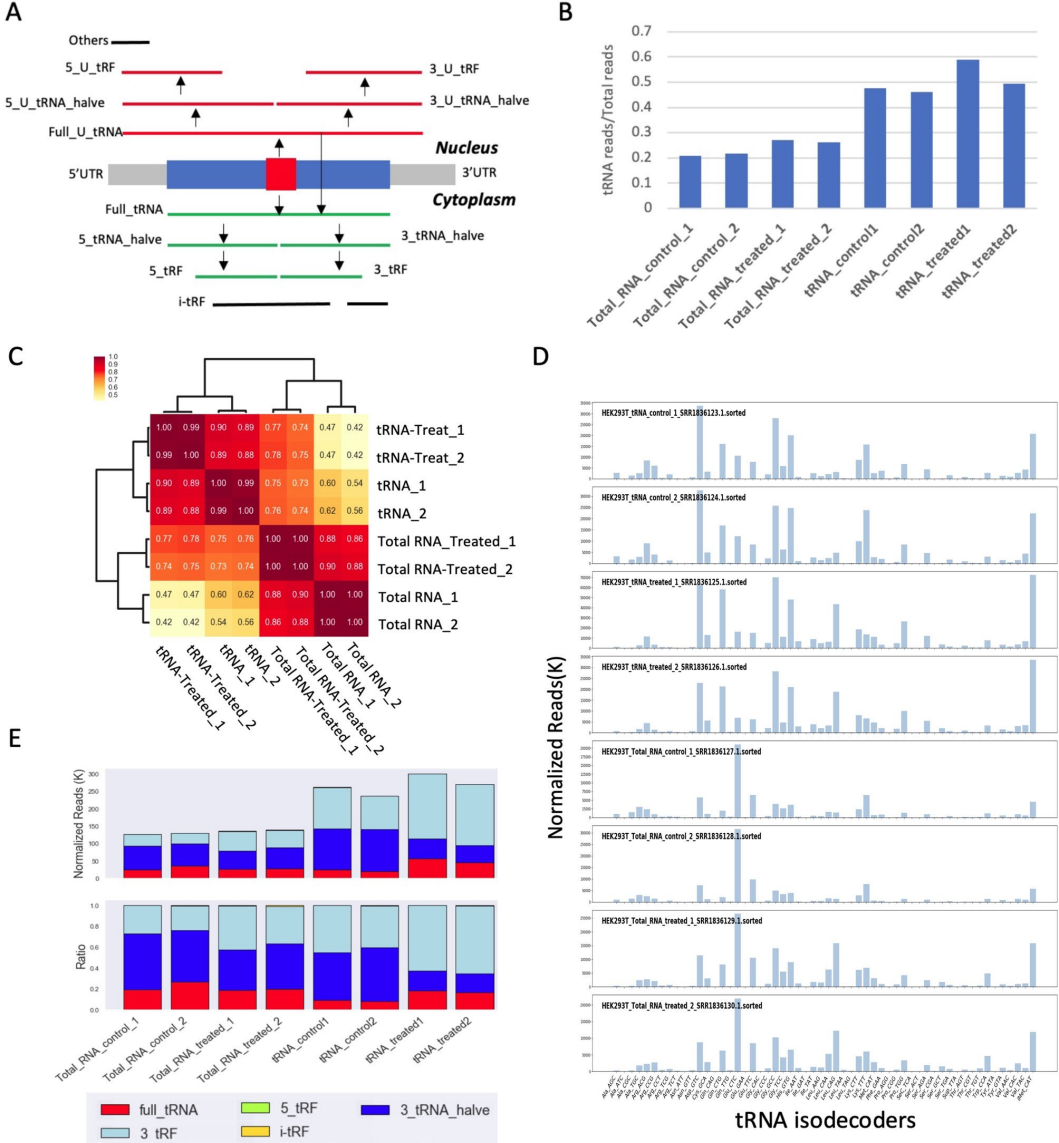
tRNA profiles of HEK293T cells in the DM-tRNA-Seq dataset. (**A**) Cartoon illustrates 12 types of tRF. The arrows represent the direction of tRNA processing; the red and dark blue rectangles in the middle represent anticodon and full-length tRNA. (**B**) Bar chart showing the proportion of tRNA-related reads in eight samples (two technical replicates for each strategy). (**C**) The cluster correlation matrix of tRNA expression profiles across all samples. The numbers in the matrix grids represent the square of the Pearson correlation coefficient (r^2^). (**D**) The expression levels (normalized read number) of 56 tRNA isodecoders in eight samples. (**E**) The normalized read counts and ratio of tRFs in the experiments. tRFs are colored as shown in the legend.

Recent studies reported an optimized small tRNA-Seq technology known as DM-tRNA-seq that combines demethylation treatment and template shift strategy to achieve better performance in the detection of full-length tRNAs^14^. We categorized tRFs into 12 types based on their mapping range on the tRNA genes (Fig. 3A). Full-length tRNAs account only for a small portion (from 14 to 23%) of tRNA pools in all samples, suggesting that tRFs, the cleavage products of mature tRNAs, are stable and may have biological significance. Consistent with the original report^14^, the proportion of tRNA-mapped reads in the cDNA libraries was significantly increased (from 20 to 50%) in the purified tRNA template compared with that in the total RNA template (Fig. 3B). The correlation and expression analyses showed that the tRNA profiles of the purified tRNA and total RNA data were dramatically different (Fig. 3C,D). For example, the correlations between the total RNA and purified tRNA data were approximately 0.42–0.76, whereas the internal correlation between these two groups was usually higher than 0.86. tRNA-Glu was the most abundant isoacceptor in the total RNA profile, which was ranked 11th in the purified tRNA profiles (Fig. 3D). These results emphasize the importance of template selection for tRNA profiling. The length distribution of tRFs in the DM-tRNA-seq dataset shows three peaks at 75 nt, 38 nt, and 57 nt, which represent full-length tRNAs, 3′-tRNA_halves, and long 3′-tRFs, respectively (Fig. S6). As expected, a greater number of long tRFs (> 50 nt) were identified in the demethylated samples, especially in the tRNA-treatment samples (Fig. S6). The relative abundance of full-length tRNAs in the tRNA-demethylated samples was higher than that in the untreated samples (Fig. 3E). A total of 16 full-length tRNA isoacceptors were identified in tRNA-demethylated samples compared with 8 identified in tRNA-untreated samples (Fig. S7). Interestingly, very few 5_tRFs, including 5_tRNA_halves, were identified in the DM-tRNA-seq dataset, which accounted for a significant proportion in the Cell Lines-Tissues samples (see below).

### Diversity of the tRF pools in the cell lines and tissues

**Figure 4:**
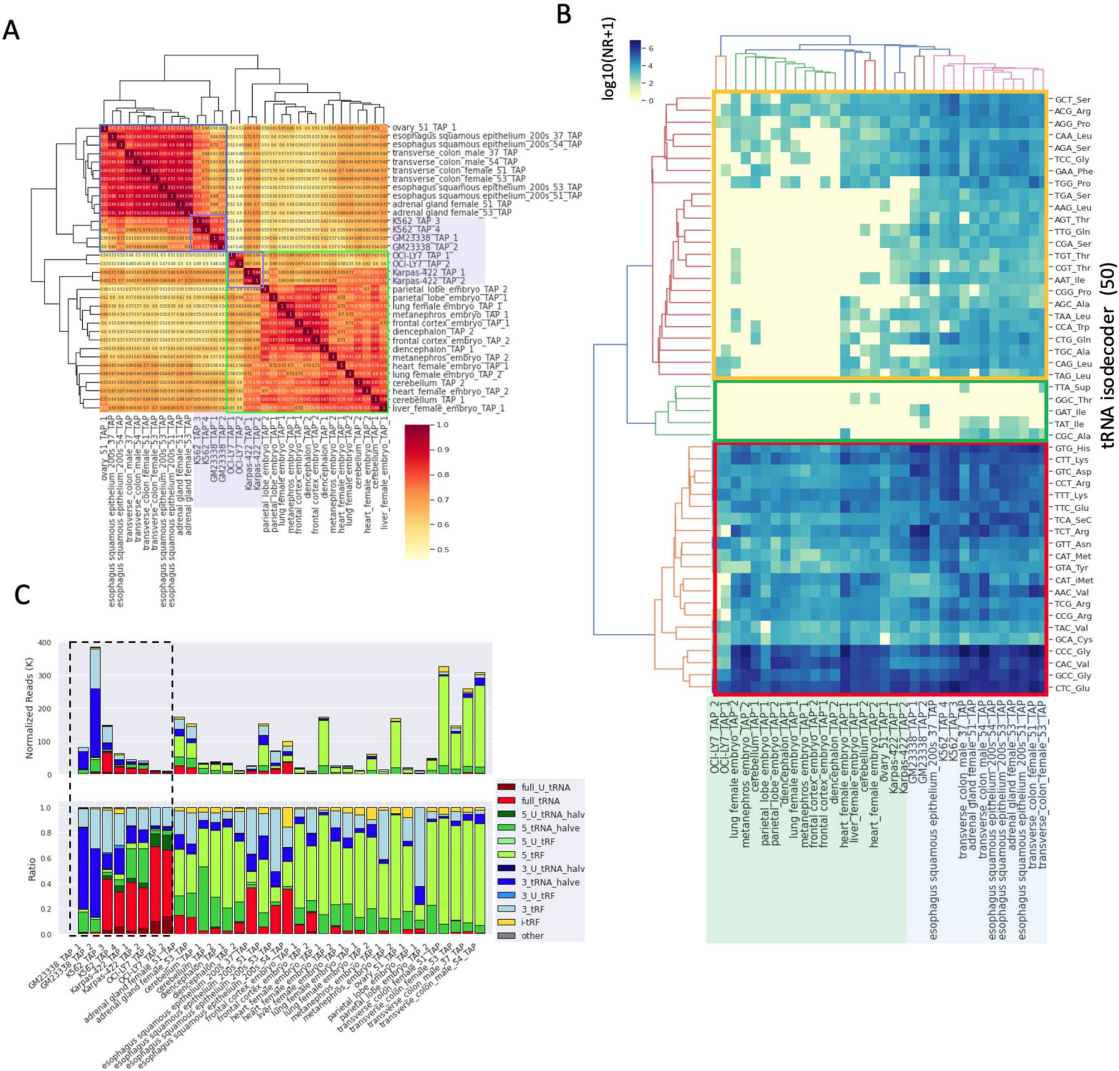
tRNA expression profiles in the cell line and tissue data. (**A**) The correlation matrix of the expression profiles of tRNA families across 34 samples. Two major clusters are highlighted by blue and green rectangles. The light purple rectangles indicate the cell line samples. (**B**) Clustered heatmap of 50 tRNA isodecoders across the samples. The expression levels of isodecoders are represented by log10(NR + 1), and the three major clusters are marked by color rectangles (red, yellow, and green rectangles represent universally expressed tRNA isodecoders, tissue-specific expressed isodecoders, and nonexpressed isodecoders, respectively). Group 1 and Group 2 samples in (**A**) are marked with light green and blue rectangles at the bottom in (**B**). (**C**) Stack bar charts showing the relative abundance and composition of tRFs in all samples. The top and bottom panels represent the relative abundance (NR) of tRNAs/tRFs and the proportions of tRFs in the samples, respectively. tRFs are colored as shown in the legend. The dot black rectangle indicates cell line samples.

tRF profiles across different samples were compared by analysis of 34 small-RNA sequencing datasets in the Cell Lines-Tissues dataset. We identified a total of 2103 tRFs, including 950 high-confidence tRFs, which were supported by at least 1000 reads in two samples (Supplemental Data Files 1 and 2). High-confidence tRFs included 3_tRFs (314), 3_tRNA_halves (199), 5_tRFs (162), and 3_tRNA_halves (199), suggesting that most tRFs are direct products of mature tRNA cleavage (Fig. S8A). The correlation analysis suggested that all samples can be clustered into two groups (blue and green rectangles in Fig. 4A). Group one included four types of tissues (ovary, esophageal squamous epithelium, transverse colon, and adrenal gland) and 2 cell lines (K562 and GM23338). The second group contained four types of tissues (parietal lobe, frontal cortex, diencephalon, metanephros, heart, liver, lung, and cerebellum) and 2 cell lines (OCI-LY7 and Karpas-422). Within each group, cell lines and tissues were separated from each other. Construction of an expression matrix of 50 tRNA isodecoders across all samples indicated that isodecoders were clustered into three groups, including 21 universally expressed tRNA isodecoders, 24 issue-specific tRNA isodecoders, and five nonexpressed tRNA isodecoders (Fig. 4B). The sample classification in the matrix was consistent with the correlation results indicating that the samples in group 2, but not group 1, express tissue-specific isodecoders. Analysis of the composition of the tRF pools in all samples indicated that 5′-tRFs and 5′-tRF-halves accounted for a significant proportion in the tissue samples but not in cell lines (Fig. 4C). In most cases, a single tRNA family had several expression patterns across the samples. For example, three tRFs were derived from the tRNA_Lys-CTT-4-1 family, including 5′-tRNA-halves, 3′-tRFs, and full-length tRNA, which were dominant in 20, 5, and 2 samples, respectively. Other 11 samples had both 5′-tRNA-halves and 3′-tRFs (Fig. S8B). Only 5′-tRNA-halves were observed in all 26 samples in the case of the tRNA-Val-TAC-4-1 family (Fig. S8C).

### 5′-Additions in tRNAs and tRFs

**Figure 5:**
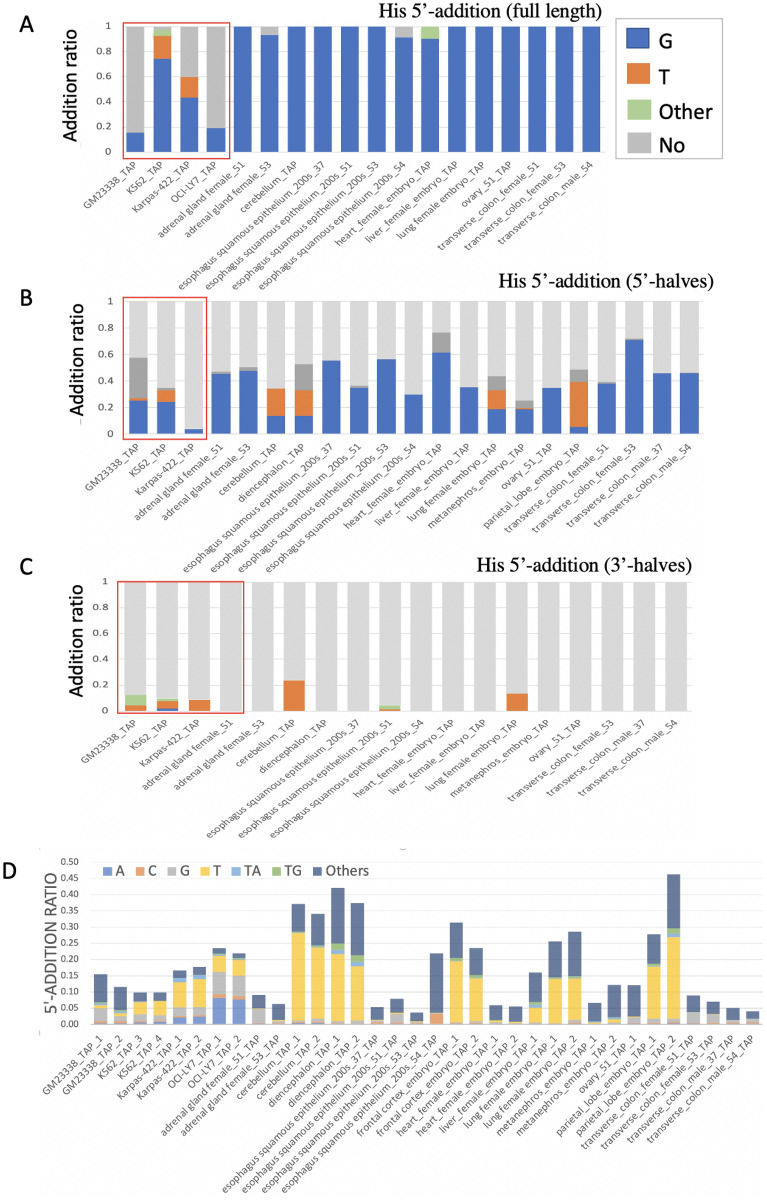
The 5′ additions of tRNAs/tRFs. (**A**–**C**) The composition of 5′-end additions of full-tRNA, 5′-tRNA-halves, and 3′-tRNA-halves of His-tRNAs, respectively, in all samples. The red boxes mark the cell lines. The types of 5′ additions are colored as shown in the legend. (**D**) Bar chart showing the composition of 5′ addition in all samples.

It is well established that the tRNA^His^ guanylytransferase (Thg1) superfamily can catalyze the 3′–5′ synthesis of nucleic acids and G_−1_ addition to tRNA^His^ with high specificity and efficiency^33^. As expected, the G_−1_ addition ratios of full-length tRNA^His^ in the tissues were above 92%, and 11 out of 13 ratios were 100%. Interestingly, compared with the tissues, cell lines had relatively lower and more diverse G_−1_ addition ratios for tRNA^His^. In K562 and Karpas-422 cells, only 73.1% and 43.1% of tRNA^His^ had G_−1_, respectively. A novel T_−1_ addition was found in 18% and 16.1% of tRNA^His^ in K562 and Karpas-422 cells, respectively. In GM23338 and OCI-LY7 cells, the ratios of G_−1_ addition were even lower (15% and 19%, respectively) (Fig. 5A). These data suggest that the Thg1 pathway may be interrupted in these cell lines. Analysis of the ratio of the 5′-halves of tRNA^His^ indicated that the G_−1_ addition ratios of the 5′-halves were significantly lower (approximately 15–70%) than that of full-length tRNA^His^ in both cell lines and tissues, with the exception of GM23338, which showed a slight increase. Moreover, T_−1_ additions were found in 5′-halves in some tissues (e.g., cerebellum, diencephalon, lung, embryo metanephros, and parietal lobe) (Fig. 5B). In the case of 3′-halves of tRNA^His^, almost no G_−1_ additions were found in any of the samples (Fig. 5C). However, T_−1_ additions were found in all three cell lines and some tissues, such as the cerebellum, esophagus, and lung. Previous reports indicates that G_−1_ addition is detected only in full-length tRNA^His^^34^. Our date suggested that the 5′-halves of tRNA^His^ may inherit G_−1_ from full-length tRNAs and gradually lose it. The presence of T_−1_ additions in 3-halves of tRNA^His^ suggested that these additions may occur after anticodon cleavage in a tissue-specific manner.

Intriguingly, novel 5′-additions (e.g., A, U, C, UG, UA, and UU) were identified in various tRNAs in many samples. Approximately 5–45% of tRFs had 5′-addition modifications, and T_−1_ addition was the most abundant modification in 18 out of 34 samples (18/24) (Fig. 5D, Supplemental Data File 3). Comparison with G_−1_ addition indicated that T_−1_ addition has lower tRNA specificity and can be detected in both full-length tRNA and 3′_tRFs, suggesting that T_−1_ addition can be added to tRFs after the maturation and cleavage of tRNAs. These patterns were detected in various samples, suggesting that they should not result from random sequencing errors or artificial bias of library construction. Overall, the data suggested that 5′-additions of tRNAs are much more complex than thought previously, and the mechanisms and functions related to these novel 5′-additions are a subject of future investigations.

### tRNA modification profiles in 293 T cells

Previous studies suggested the potential use of NGS technology to identify and quantify tRNA base methylation^35, 36^. Using tRNAExplorer, we reanalyzed the DM-tRNA-Seq dataset and found that the treatment with the dealkylating enzyme AlkB, which removes the methyl groups from m^1^A and m^3^C to revert the bases to their unmodified forms, dramatically decreased the ratio of mismatched reads vs total reads from approximately 89% (tRNA_control) to 31% (tRNA_treated), suggesting that most of the mismatches in the tRNA sequencing data may result from tRNA methylation (Fig. S9A). We calculated the mismatch ratio (MR) for all mismatched sites and found two peaks in the MR distribution at approximately 0% and 100% in all samples; AlkB treatment removed most of mismatched sites with MR between the two peaks (Fig. S9B). We identified a total of 16 potential modification sites (MR > 80% and > 200 supporting reads) across 44 isoacceptors in the tRNA-contol and tRNA-treatment data (Table S5).

**Figure 6:**
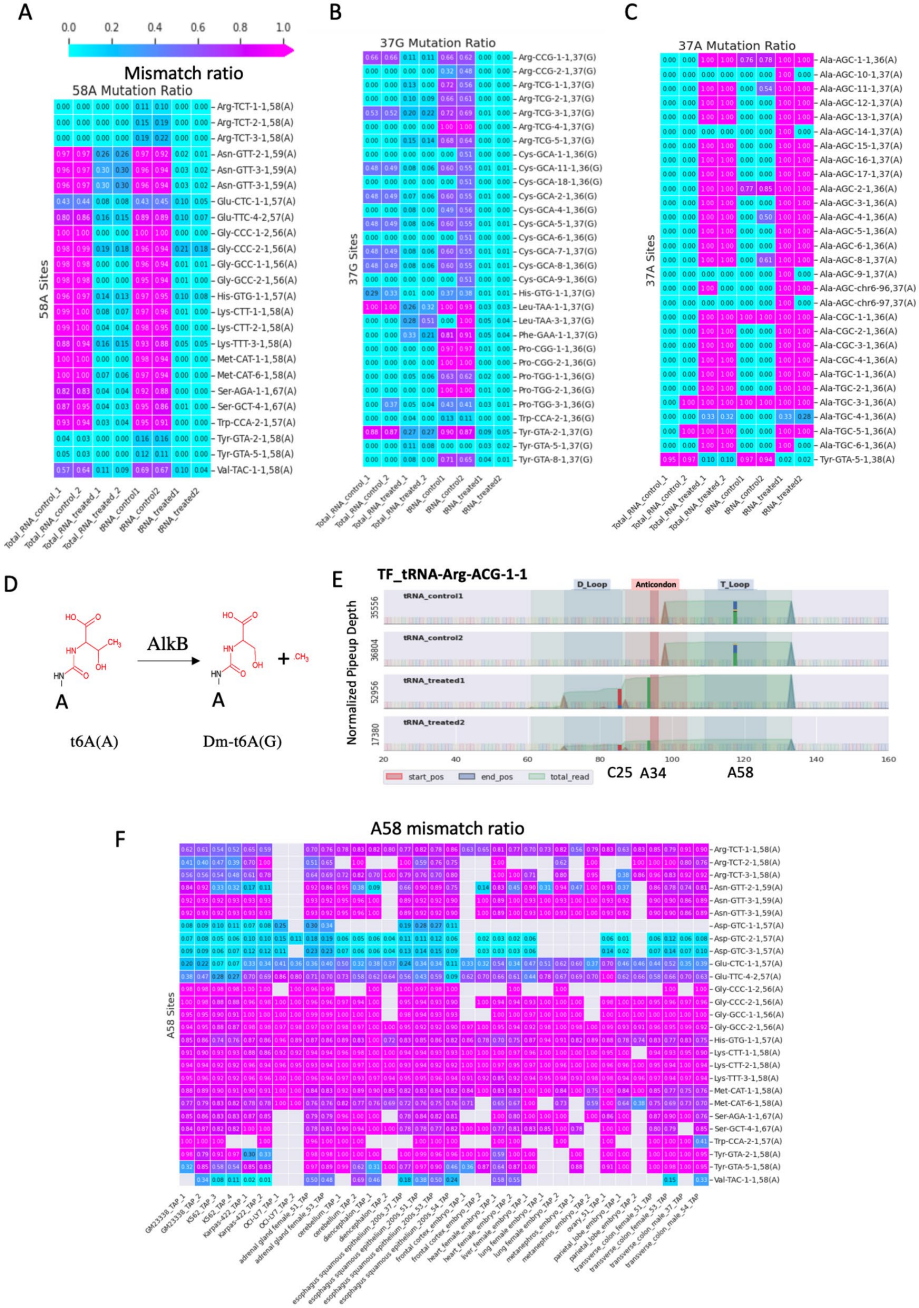
tRNA modifications identified in the DM-tRNA-seq and Cell Lines-Tissues datasets. (**A**–**C**) Matrixes of mismatch ratios of A58, 37G, and 37A in the DM-tRNA-seq dataset. The mismatch ratios are shown in the units that according to the colors indicated in the legend bar. (**D**) The reaction of methyl group elimination from t6A by AlkB. (**E**) tRF profile of the tRNA-Arg-ACG-1-1 family shows three potential modification sites (C25, A34, and A58). Small bars show the locations and proportion of mismatches. (**F**) The mismatch ratios of A58 of 27 tRNA families in the samples of the Cell Lines-Tissues dataset.

The 58A mismatches were the most popular and were detected in most of the isoacceptors (41/44). These mismatches were detected only in tRNA-controls and were absent from the tRNA-treatment (AlkB) samples, suggesting the existence of m^1^A modification (Fig. 6A). This result is consistent with previous reports that demonstrated the presence of m^1^A58 in almost all human tRNAs^35^. We also found that the modification ratios of m^1^A58 were relatively low in the Arg-TCT, Glu-CTC, and Tyr-GTA tRNA families (Fig. 6A).

We also identified mismatches at position 34 in the tRNA nomenclature (wobble base of anticodon) in both tRNA-control and tRNA-treatment samples (Fig. S9C). The A-to-inosine (I) modification at the wobble base was reported in at least eight human tRNAs, which may be important for expanding the base-pairing capability from A34-U to I34-U/I34-C and even I34-A^37^. Our results show that approximately seven isoacceptors have this modification in 293 T cells; the mismatch ratios are always 100% and are not influenced by AlkB treatment (Fig. S9D). Although 2′O-methylation of G34/C34 is another common wobble modification in human tRNAs, we did not detect the G34/C34 mismatches, suggesting that NGS sequencing may not have sufficient sensitivity to detect this modification.

Nucleotide 37 in tRNAs is an A or G and is located at the immediate 3′ end of the anticodon nucleotides. Modifications of this position, such as N^1^-methylguanosine (m^1^G), N^6^-threonylcarbamoyladenosine (t^6^A), and N^6^-isopentenyladenosine (i^6^A), were reported to be essential for the prevention of frameshifting^38^. We identified the 37G and 37A mismatches in 7 and 3 isoacceptors, respectively (Fig. 6B,C). Interestingly, the 37A (A- > G) mismatches were detected only in the AlkB-treated samples, indicating that AlkB treatment may remove the methyl group from t^6^A or i^6^A thus enabling the detection of these modifications by NGS sequencing (Fig. 6D). As expected, the 37G modifications should be m^1^G because mismatches were only observed in the tRNA-control samples and were absent from the tRNA-treatment samples.

The m^2^_2_G26 modification between the D and anticodon stems is assumed to be important for an increase in the stiffness of tRNAs^39^. We identified 19 isoacceptors that may have this modification in 293 T cells (Table S5). Many G26 mismatches were observed in both control and AlkB treatment samples, suggesting that m^2^_2_G26 modification is resistant to AlkB treatment. The absence of G26 mismatches in some control samples is primarily due to the lack of the reads covering G26.

Additionally, ten mismatch sites, including A52, A67, A69, C25, G56, C32, C51, U53, U27, and U28, were observed only in one or two isoacceptors; C32 is known as a 3′-methylation site^36^. The functions and modification types of other sites are largely unknown. Overall, our results suggest the potential use of mismatches in the tRNA-Seq data to identify tRNA modifications and quantify their ratios in human samples (Fig. 6E, Table S5).

### Modification profiles across tissues and cell lines

To investigate the general patterns of tRNA modification across the samples and identify tissue/cell line-specific modification events, we calculated the mismatch ratio using the tissue and cell line data. The matrix of 58A tRNA families across all samples indicated that the modification status of A58 is distinct between the tRNA families. Asp-GTC-tRNAs and Val-TAC-tRNAs had the lowest modification ratios (2–30%); Glu-CTC-tRNAs, Glu-TTC-tRNAs, and Val-TAC-tRNAs had moderate modification levels (10–70%), and other families had high modification levels (70–100%) (Fig. 6F). In the case of the G9 (m^1^G) sites, the modification ratio in Val-TAC-4-1 was usually approximately 100%, which was significantly higher than that in other families. On the other hand, the G9 modification ratios in Glu-TTC, Glu-CTC, Pro-AGG, and Pro-CGG tRNAs were usually less than 10% in most samples. The data also indicated some tissue-specific modification events (Fig. S10A). For example, we identified four potential modification sites (G7, G10, A57, and G64) in most of the samples in the tRNA-Glu-CTC family, which is the most abundant tRNA family. In the case of A57, the modification ratios across the samples range from 7% (K562 cells) to 70% (ovary), suggesting that the modification statuses of the site may vary in various samples. However, the distributions of three other modifications were generally uniform (Fig. S10B).

### The cleavage sites of tRNAs

tRFs are the products of cleavage of tRNAs; hence, it is possible to detect the cleavage events based on the start and end sites of tRFs. However, it is necessary to distinguish true cleavage sites from early stops of cDNA synthesis triggered by certain posttranscriptional modifications. In the DM-tRNA-Seq dataset, cDNAs were synthesized by template switching from the adaptor to the 3′ end of the target RNA. In the case of the 5′ ends of tRFs, it is almost impossible to distinguish the cleavage sites from the early stop sites. On the other hand, the 3′ ends of tRFs should result from tRNA cleavage. However, most tRFs (> 99%) end with the 3′-termini of the tRNA genes (Fig. 3E) in the datasets; thus, no new cleavage sites can be identified based on the data.

**Figure 7:**
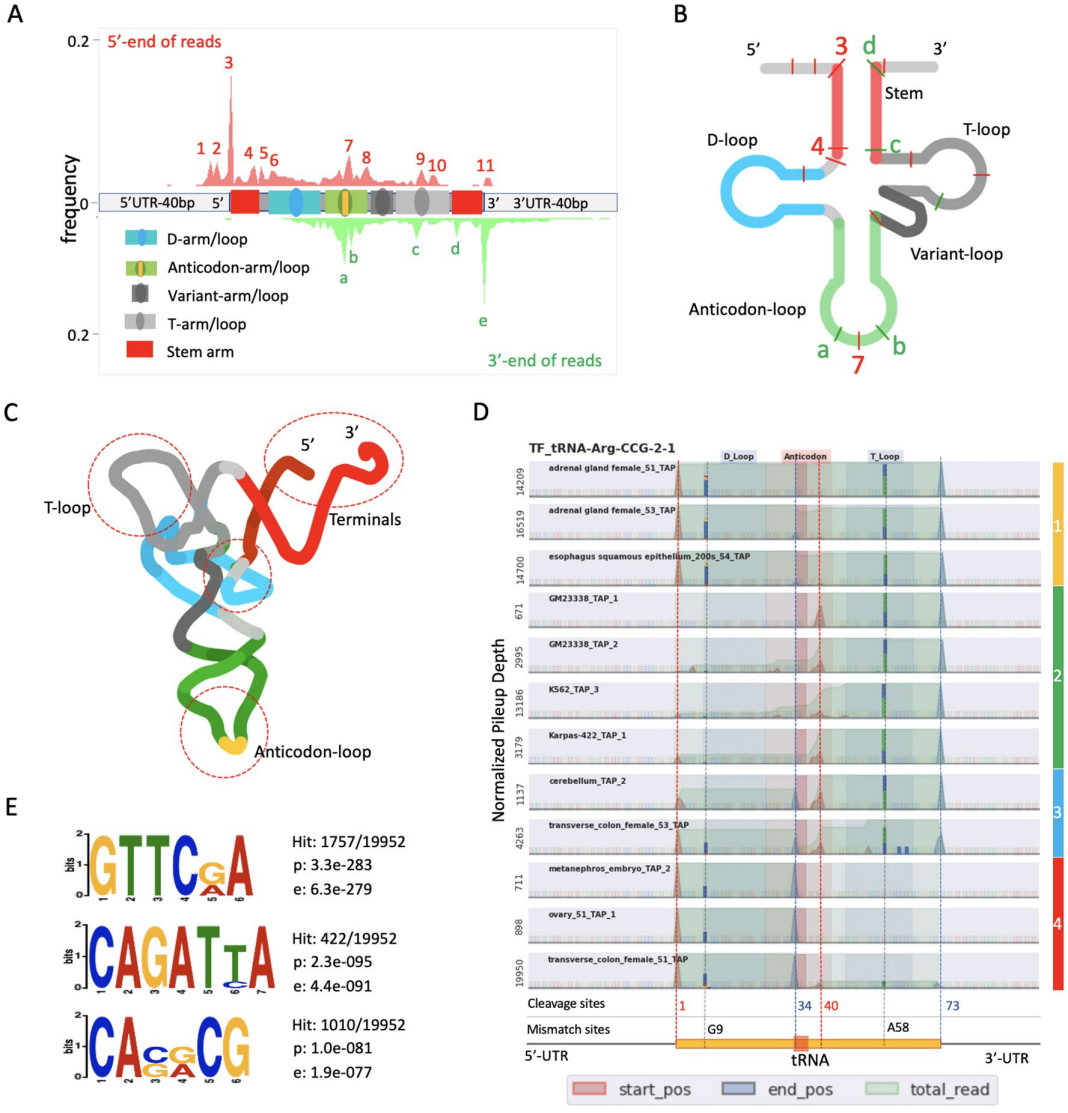
The cleavage sites of tRNAs. (**A**) The accumulated distribution of the cleavage sites on a virtual tRNA. The red and green area plots denote the accumulated frequencies of the 5′ and 3′ ends of tRFs (including ± 40 bp from these regions) in all samples. The arms and loops of tRNAs are shown as indicated in the legend. The cleavage hot spots are labeled as numbers (5′-end) and letters (3′-end). (**B**) Cartoon representation of the tRNA secondary structure with all cleavage hot spots labeled in Fig. 6A. (**C**) Cartoon representation of tRNA 3D structure with the hot cleavage regions highlighted by dotted circles; (**D**) The pileup profiles of tRNA-His-CCG-2-1 in 12 samples. The red and green peaks and dotted lines indicate the mapping locations of the 5′ ends and 3′ ends of the reads and potential cleavage sites, respectively. Light green area plots (total reads) are the pileup profiles of the tRNA-related reads. The numbers on the Y-axis represent the normalized pileup depth. The regions of the tRNA body and anticodon are represented by orange and light-red blocks. The color bars on the right side indicate the classes of RNA-Seq profiles. (**E**) The top three motifs enriched in the tRNA cleavage sites identified by the DREME tool. Hits correspond to the ratio of matched sequences to the total number of sequences. The p-value corresponds to the p-value of Fisher’s exact test for enrichment of a motif in the positive sequences, and e is the motif p-value times the number of candidate motifs tested.

In the Cell Lines-Tissues dataset, a poly-A tail and 5′-RNA adapter were added at the 3′ and 5′ ends of RNAs, respectively, before cDNA synthesis (https://www.encodeproject.org/experiments/ENCSR000CRF/). Early cDNA synthesis stop events can result in the absence of a 5′-RNA adapter of the first strand of cDNA and the failure of cDNA amplification (loss of tRFs) but do not result in the truncation of tRFs. Therefore, both the 5′ and 3′ ends of tRFs observed in the Cell Lines-Tissues dataset should result from RNA cleavage or random degradation, which can be distinguished by frequency. We identified a total of 1027 cleavage sites that were supported by at least 1000 reads in two samples (Supplemental Data File 4). We mapped the peaks of the 5′ and 3′ ends of tRFs to a virtual tRNA 70 bp in length to calculate the cleavage frequencies for each base in the virtual tRNA. The results indicated the presence of 11 and 5 peaks of cleavage sites suggested by the 5′ and 3′ ends, respectively (Fig. 7A). Most of the sites were located within five regions (5′ and 3′ terminals of tRNA, the connection region between the stem arm and D-loop, the anticodon loop, and the T-loop) (Fig. 7B). The 3D structure of tRNA suggests that all regions form exposed surfaces that are easily accessed by RNases (Fig. 7C). In addition to the 3′ and 5′ ends of mature tRNAs, cleavage sites are enriched within the anticodon loop or even in the anticodon itself (peaks a, b, and 7 in Fig. 7A). These sites are close to the wobble base (34) and 37 A/G. Additionally, the pileup profiles of TF-tRNA-Arg-CCG-2-1 can be categorized into four groups. Two conserved cleavage sites (T33/C34 and C40/A41) were found in groups 2, 3, and 4 and resulted in the production of 5′-tRNA-halves of a 3′-tRF (Fig. 7D). To explore the sequence specificity of the cleavage sites, we extracted 19952 unique sequences flanking high-confidence cleavage sites (supported by a minimum of 100 reads) with 7 bases and found 110 enriched patterns (Fig. 7E and Supplemental Data File 5).

## Discussion

In this study, we systematically examined the enrichment of eight histone modifications and 248 DNA-binding factors in the tRNA genes in three human cell lines to explore the mechanisms that regulate the tRNA pathway at the transcriptional level. As expected, active tRNA genes were consistently enriched with active markers (such as H3K4me3 and H3K4me2) and did not have repressive markers (such as H3K9me3 and H3K27me3) in various cells, suggesting that histone methylation may serve as a common mechanism that enables the expression of the tRNA genes. Interestingly, our data showed that highly expressed tRNA genes may serve as hubs of trans-factor binding and can recruit both transcriptional activators and repressors. For example, many subunits of histone acetylation and deacetylation complexes bind to and may compete for the tRNA genes in a cell-specific manner. Additionally, several signaling pathways (for example, the TORC1, Myc, ERK, and p53 pathways) are known to be involved in the Pol-III and tRNA synthesis systems^40–42^. Our study added several new candidates to the list, including the cAMP pathway in the tumor cell lines and the pluripotent pathway in H1 embryonic stem cells. These pathways are linked to human diseases and organism development^27, 32^; thus, our observations suggest the possible crosstalk between these pathways and present new clues for the investigations of novel tRF functions.

Additionally, our study compared tRF profiles across five human cell lines and eight tissues. The datasets used in the present study have some limitations (see below); however, the results clearly show the potential use of small-RNA NGS sequencing technology as a powerful method to simultaneously monitor the expression and modification, addition, and cleavage events of tRNA. Previously, several tRF databases were published^12, 21, 23^; however, most of them were based on short RNA-sequencing data (read length < 40 nt), which complicates identification of the full sequences of long tRFs. The results of the present study indicated that a large portion of tRFs (50–60%) were longer than 40 nt (Fig. S6); therefore, we used long RNA-sequencing data (read length ≥ 100 nt) to avoid these issues. Our study presents a high-confidence tRF list for further investigation (Supplemental Data File 1). The data indicate that full-length tRNAs comprise only a small proportion of tRNA pools, suggesting the biological significance of tRFs. The compositions of tRFs were diverse across the samples, suggesting the existence of tissue/cell line-specific regulatory mechanisms. Our analyses also identified new modification sites and new patterns of modification and present valuable data resources to explore the tRNA dynamics. For example, the observation that the ratio of G_−1_ addition of tRNA^His^ in cell lines is significantly lower than that in normal tissues suggests a dysfunction of the Thg1 pathway in the cell line samples. Additionally, our study identified more than 100 highly enriched sequence motifs flanking the tRNA cleavage sites (Fig. 7E). Although very few RNases were determined to have sequence specificity, our results suggest that some tRNA-cleaving RNases may recognize specific sequences or, interestingly, can use tRFs as guide RNAs to target cleavage sites similar to CRISPR. It is notable, that many clues and hypothesis raised by our study need be further validated by additional benchworks and data analyses.

Although optimized experimental methods, such as DM-tRNA-Seq and ARM-Seq, have significantly improved the sensitivity of tRNA sequencing technology, there is considerable room for improvement^14, 15^. For example, very few 5-tRNA-halves were detected in the DM-tRNA-Seq dataset (Fig. 3E), suggesting the significant 3′-bias of RNA sequencing. HEK293T libraries were generated using a template switch method^14^; thus, whether the absence of 5-tRNA-halves/5′-tRFs results from 3′-sequencing bias or the true biological nature of HEK293T cells requires additional investigation. Furthermore, cDNAs undergoing early RT stop can be amplified by the template switch method, which makes it difficult to distinguish tRNA cleavage events from the early stop of RT. Additionally, AlkB treatment can remove only limited modifications (N^1^-methyladenosine (m^1^A), N^3^-methylcytidine (m^3^C) and N^1^-methylguanosine (m^1^G))^15^. Combination with other enzymes may improve the performance. Our analyses indicate that many modifications (for example, m^1^A58) do not induce early stops. Therefore, enzyme treatment should focus on the modifications that induce early stops. In the case of the Cell Lines-Tissues samples, regular A-tailing cDNA requires adaptor ligation at both ends for cDNA amplification and therefore may result in the loss of tRNA/tRFs due to early stops, which may be triggered by the modifications or secondary structure of tRNAs. The tRNA-Seq methods should be optimized in the following directions: (1) using purified tRNA rather than total RNA as template; (2) using ligation-based library construction method to avoid missing 5′_tRFs and 5′_tRNA_halves and inability to discriminate RNA cleavage from an early stop of RT; (3) removing more modifications that induce an early stop; (4) parallel experiments with and without enzyme treatments are required to evaluate the treatment efficiency and identify new modification sites; (5) the read length of RNA-Seq should be above 100 bp to capture long tRFs; and (6) advanced bioinformatics tools, such as tRNAExplorer, are required for simultaneous evaluation of tRNA/tRF quantification, modification, addition, and cleavage.

## Conclusion

In summary, we extensively investigated the regulatory mechanisms and landscapes of tRNA using 1332 Chip-Seq datasets and 36 small-RNA-Seq datasets from human cell lines and tissues and found that tRNA genes are regulated by distinct transcriptional regulators, including histone acetylation, cAMP, and pluripotency pathways. We also identified 950 high-confidence tRFs suggesting that tRNA pools are dramatically distinct across cells and tissues in terms of the expression profiles and tRF composition. Tissues usually have higher levels of 5′-tRFs than that in cell lines. The analysis identified new potential modifications and uncovered specific cleavage patterns in tRNA families. The results also showed that RNA library preparation technologies have a considerable impact on tRNA profiling and require optimization in the future. Overall, our study provides a comprehensive global view and new insight into tRNA pools across various human cell lines and tissues.

## Supplementary Information

Supplementary Information.

Supplementary Data.

## Data Availability

*Accession numbers* The accession number list of the ChIP-Seq and RNA-Seq data used in this paper can be found in “Supplemental materials” (Datas S9–S13).

## Abbreviations

tRFs: Small non-coding RNA derived from tRNAs, it contains 12 categories including full-length tRNAs in the paper
DBP: DNA binding proteins
PCR: Polymerase chain reaction

## References

[1] Grewal SS (2015). Why should cancer biologists care about tRNAs? tRNA synthesis, mRNA translation and the control of growth. Biochim. Biophys. Acta Gene Regul. Mech..

[2] Blanco S, Bandiera R, Popis M (2016). Stem cell function and stress response are controlled by protein synthesis. Nature.

[3] Rapino F, Delaunay S, Zhou Z, Chariot A, Close P (2017). tRNA modification: Is cancer having a wobble?. Trends Cancer..

[4] Shigematsu, M., Honda, S., Kirino, Y. Transfer RNA as a source of small functional RNA. *J. Mol. Biol. Mol. Imaging*. 1(2) (2014). http://www.ncbi.nlm.nih.gov/pubmed/26389128. Accessed 12 February 2019. PMC4572697 26389128

[5] Selitsky SR, Baran-Gale J, Honda M (2015). Small tRNA-derived RNAs are increased and more abundant than microRNAs in chronic hepatitis B and C. Sci. Rep..

[6] Goodarzi H, Liu X, Nguyen HCB, Zhang S, Fish L, Tavazoie SF (2015). Endogenous tRNA-derived fragments suppress breast cancer progression via YBX1 displacement. Cell.

[7] Shigematsu M, Kirino Y (2015). tRNA-derived short non-coding RNA as interacting partners of argonaute proteins. Gene Regul. Syst. Biol..

[8] Sharma U, Conine CC, Shea JM (2016). Biogenesis and function of tRNA fragments during sperm maturation and fertilization in mammals. Science.

[9] Balatti V, Nigita G, Veneziano D (2017). tsRNA signatures in cancer. Proc. Natl. Acad. Sci..

[10] Goodarzi H, Nguyen HCB, Zhang S, Dill BD, Molina H, Tavazoie SF (2016). Modulated expression of specific tRNAs drives gene expression and cancer progression. Cell.

[11] Schimmel P (2018). RNA processing and modifications: The emerging complexity of the tRNA world: Mammalian tRNAs beyond protein synthesis. Nat. Rev. Mol. Cell Biol..

[12] Kumar P, Mudunuri SB, Anaya J, Dutta A (2015). tRFdb: A database for transfer RNA fragments. Nucleic Acids Res..

[13] Chan PP, Lowe TM (2016). GtRNAdb 2.0: An expanded database of transfer RNA genes identified in complete and draft genomes. Nucleic Acids Res..

[14] Zheng G, Qin Y, Clark WC (2015). Efficient and quantitative high-throughput tRNA sequencing. Nat. Methods..

[15] Cozen AE, Quartley E, Holmes AD, Hrabeta-Robinson E, Phizicky EM, Lowe TM (2015). ARM-seq: AlkB-facilitated RNA methylation sequencing reveals a complex landscape of modified tRNA fragments. Nat. Methods..

[16] Gogakos T, Brown M, Garzia A, Meyer C, Hafner M, Tuschl T (2017). Characterizing expression and processing of precursor and mature human tRNAs by hydro-tRNAseq and PAR-CLIP. Cell Rep..

[17] Torres AG, Reina O, Attolini CSO, De Pouplana LR (2019). Differential expression of human tRNA genes drives the abundance of tRNA-derived fragments. Proc. Natl. Acad. Sci. USA.

[18] Sloan CA, Chan ET, Davidson JM (2016). ENCODE data at the ENCODE portal. Nucleic Acids Res..

[19] Ramírez F, Ryan DP, Grüning B (2016). deepTools2: a next generation web server for deep-sequencing data analysis. Nucleic Acids Res..

[20] Loher P, Telonis AG, Rigoutsos I (2017). MINTmap: Fast and exhaustive profiling of nuclear and mitochondrial tRNA fragments from short RNA-seq data. Sci. Rep..

[21] Pliatsika V, Loher P, Magee R (2018). MINTbase v2.0: A comprehensive database for tRNA-derived fragments that includes nuclear and mitochondrial fragments from all the Cancer Genome Atlas projects. Nucleic Acids Res..

[22] Pliatsika V, Loher P, Telonis AG, Rigoutsos I (2016). MINTbase: A framework for the interactive exploration of mitochondrial and nuclear tRNA fragments. Bioinformatics.

[23] Zheng LL, Xu WL, Liu S (2016). tRF2Cancer: A web server to detect tRNA-derived small RNA fragments (tRFs) and their expression in multiple cancers. Nucleic Acids Res..

[24] Bolger AM, Lohse M, Usadel B (2014). Trimmomatic: A flexible trimmer for Illumina sequence data. Bioinformatics.

[25] Franceschini A, Szklarczyk D, Frankild S (2013). STRING v9.1: Protein-protein interaction networks, with increased coverage and integration. Nucleic Acids Res..

[26] Bateman A, Martin MJ, O’Donovan C (2015). UniProt: A hub for protein information. Nucleic Acids Res..

[27] Verstrepen KJ, Derdelinckx G, Dufour JP (2003). The Saccharomyces cerevisiae alcohol acetyl transferase gene ATF1 is a target of the cAMP/PKA and FGM nutrient-signalling pathways. FEMS Yeast Res..

[28] Gozdecka M, Breitwieser W (2012). The roles of ATF2 (activating transcription factor 2) in tumorigenesis. Biochem. Soc. Trans..

[29] Daly NL, Arvanitis DA, Fairley JA (2005). Deregulation of RNA polymerase III transcription in cervical epithelium in response to high-risk human papillomavirus. Oncogene.

[30] White RJ (2004). RNA polymerase III transcription and cancer. Oncogene.

[31] Chew J-L, Loh Y-H, Zhang W (2005). Reciprocal transcriptional regulation of Pou5f1 and Sox2 via the Oct4/Sox2 complex in embryonic stem cells. Mol. Cell Biol..

[32] Fogarty NME, McCarthy A, Snijders KE (2017). Genome editing reveals a role for OCT4 in human embryogenesis. Nature.

[33] Williams JB, Cooley L, Söll D (1990). Enzymatic addition of guanylate to histidine transfer RNA. Methods Enzymol..

[34] Rao BS, Maris EL, Jackman JE (2011). TRNA 5′-end repair activities of tRNAHis guanylyltransferase (Thg1)-like proteins from Bacteria and Archaea. Nucleic Acids Res..

[35] Clark WC, Evans ME, Dominissini D, Zheng G, Pan T (2016). tRNA base methylation identification and quantification via high-throughput sequencing. RNA.

[36] Pan T (2018). Modifications and functional genomics of human transfer RNA. Cell Res..

[37] Novoa EM, Pavon-Eternod M, Pan T, De Pouplana LR (2012). A role for tRNA modifications in genome structure and codon usage. Cell.

[38] Schweizer U, Bohleber S, Fradejas-Villar N (2017). The modified base isopentenyladenosine and its derivatives in tRNA. RNA Biol..

[39] Liu J, Straby KB (2000). The human tRNA(m22G26)dimethyltransferase: Functional expression and characterization of a cloned hTRM1 gene. Nucleic Acids Res..

[40] Marshall L, Rideout EJ, Grewal SS (2012). Nutrient/TOR-dependent regulation of RNA polymerase III controls tissue and organismal growth in Drosophila. EMBO J..

[41] Dai MS, Sun XX, Lu H (2010). Ribosomal protein L11 associates with c-Myc at 5 S rRNA and tRNA genes and regulates their expression. J. Biol. Chem..

[42] Felton-Edkins ZA, Kenneth NS, Brown TRP (2003). Direct regulation of RNA polymerase III transcription by RB, p53 and c-Myc. Cell Cycle.

