# Supplementary material for: Integrated genomic analysis reveals regulatory pathways and dynamic landscapes of the tRNA transcriptome
Source: Sci Rep. 2021 Mar 4;11:5226. doi: 10.1038/s41598-021-83469-6 (PMC7933247; doi:10.1038/s41598-021-83469-6)
Supplement: Supplementary file 1 — Supplementary Information. [file 41598_2021_83469_MOESM1_ESM.docx]

**Supplemental Materials**

**Integrated genomic analysis reveals regulatory pathways and dynamic landscapes of the tRNA transcriptome**

Zefang Sun^1^, Jia Tan^1^, Minqiong Zhao^1^, Qiyao Peng^1^, Mingqing Zhou^1^, Shanru Zuo^1^, Feilong Wu^1^, Xueguang Li^1^, Yangyang Dong^1^, Ming Xie^1^, Yide Yang^1^, Junhua Zhou^1^, Xianghua Liu^2^, Quanze He^3^, Zuping He^1^, Xing Yu^1^, Quanyuan He^1*^

**Figures**

**Figure S1.** The data processing and analyses workflow of this study.

**Figure S2.** The ChIP-Seq profiles of 243 DNA binding proteins (DBPs) and 10 histone modifications on tRNA genes in K562 cell.

**Figure S3.** The ChIP-Seq profiles of 155 DNA binding proteins (DBPs) and 9 histone modifications on tRNA genes in Hepg2 cell.

**Figure S4** The ChIP-Seq profiles of 83 DNA binding proteins (DBPs) and 6 histone modifications on tRNA genes in H1-ES cell.

**Figure S5.** Differential histone epigenetic markers and DBPs profiles on the tRNA genes in K562 and Hepg2 cells.

**Figure S6.** The distribution of tRFs’ length in DM-tRNA-Seq data.

**Figure S7.** The tRF composition of tRNA isodecoders in DM-tRNA-Seq data.

**Figure S8.** tRFs distribution and tRF profiles in Cell Lines-Tissues data.

**Figure S9.** The mismatches and modifications in DM-tRNA-Seq data.

**Figure S10.** The pattern of mismatches in Cell line and Tissue data.

**Tables**

**Table S1:** The hierarchical classification of tRNA genes and their fragments in human genome

**Table S2.** Top tRNA gene binding proteins/genes in HepG2

**Table S3.** Top tRNA gene binding proteins/genes in K562

**Table S4.** Top tRNA gene binding proteins/genes in H1-ES cell line

**Table S5.** Mismatch sites identified in the DM-tRNA-Seq data.

**Data Files (supp_data_files.zip)**

1. **1_High_Confid_tRFs.xlsx:** High confident tRF list and expression matrix.
2. **2_Full_tRFs.xlsx:** Full list of tRFs and expression matrix.
3. **3_Addition_5_data.xlsx:** The statistic of 5’-Addition of tRFs in Cell Lines-Tissues data.
4. **4_Cleavage_Sites.tsv:** The statistic of cleavage sites in Cell Lines-Tissues data.
5. **5_DREME_Motif.pdf**: DREME report for the sequence Motifs on tRNA Cleavage sites
6. **6_Hepg2_tRNA_binding_matrix.txt:** Tab file for ChIP data of Hepg2 cell used in Figure 1A.
7. **7_K562_tRNA_binding_matrix.txt:** Tab file for ChIP data of K562 cell used in Figure 1B.
8. **8_H1-ES_tRNA_binding_matrix.txt:** Tab file for ChIP data of H1-ES cell used in Figure S4.
9. **9_DM-tRNA-Seq_static.tsv :** Tab file for DM-tRNA-Seq dataset.
10. **10_Cellline_Tissues_Static.tsv:** Tab file for human Cellline_Tissues dataset.
11. **11_H1_ESCs_CHIP_Seq_data_source.xls :** Tab file for 94 ChIP-Seq data for H1-ESC cell
12. **12_HepG2_CHIP_Seq_data_source.xls :** Tab file for 528 ChIP-Seq data for Hepg2 cell
13. **13_K562_CHIP_Seq_data_source.xls :** Tab file for 713 ChIP-Seq data for K562 cell
14. **14_K562_pro_full_tRNA_binding_matrix.txt:** Tab/Matrix file for ChIP data of K562 cell used in Figure S1
15. **15_HepG2_full_pro_tRNA_binding_matrix.txt:** Tab/Matrix file for ChIP data of Hepg2 cell used in Figure S2
16. **16_HepG2_K562_Top_tRNA_gene_binding_proteins_tab.xls:** List of genes binding to tRNA genes in HepG2 and K562.

**Figure S1**

**
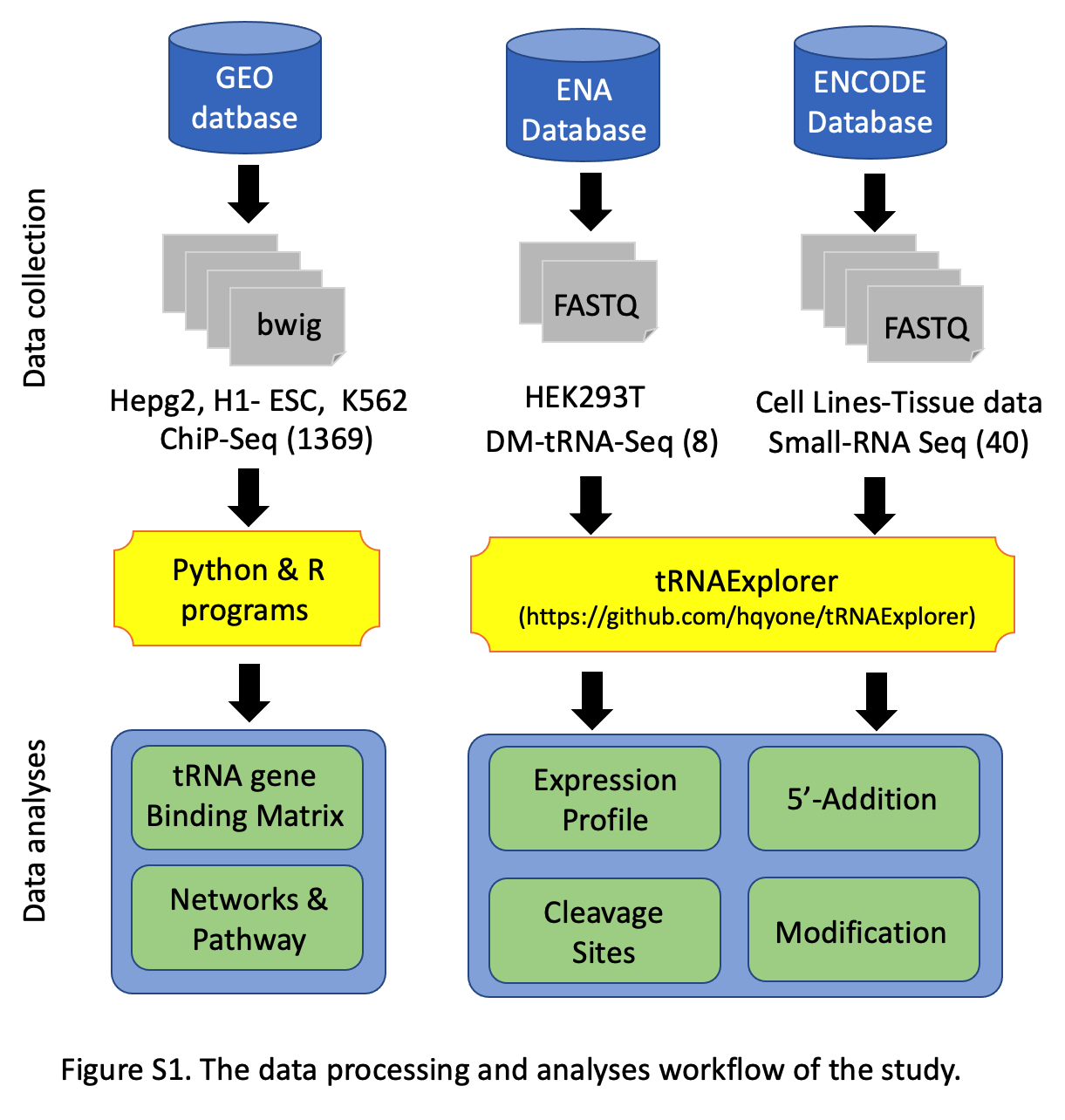
**

**Figure S1. The data processing and analyses workflow of the study.**

**Figure S2**


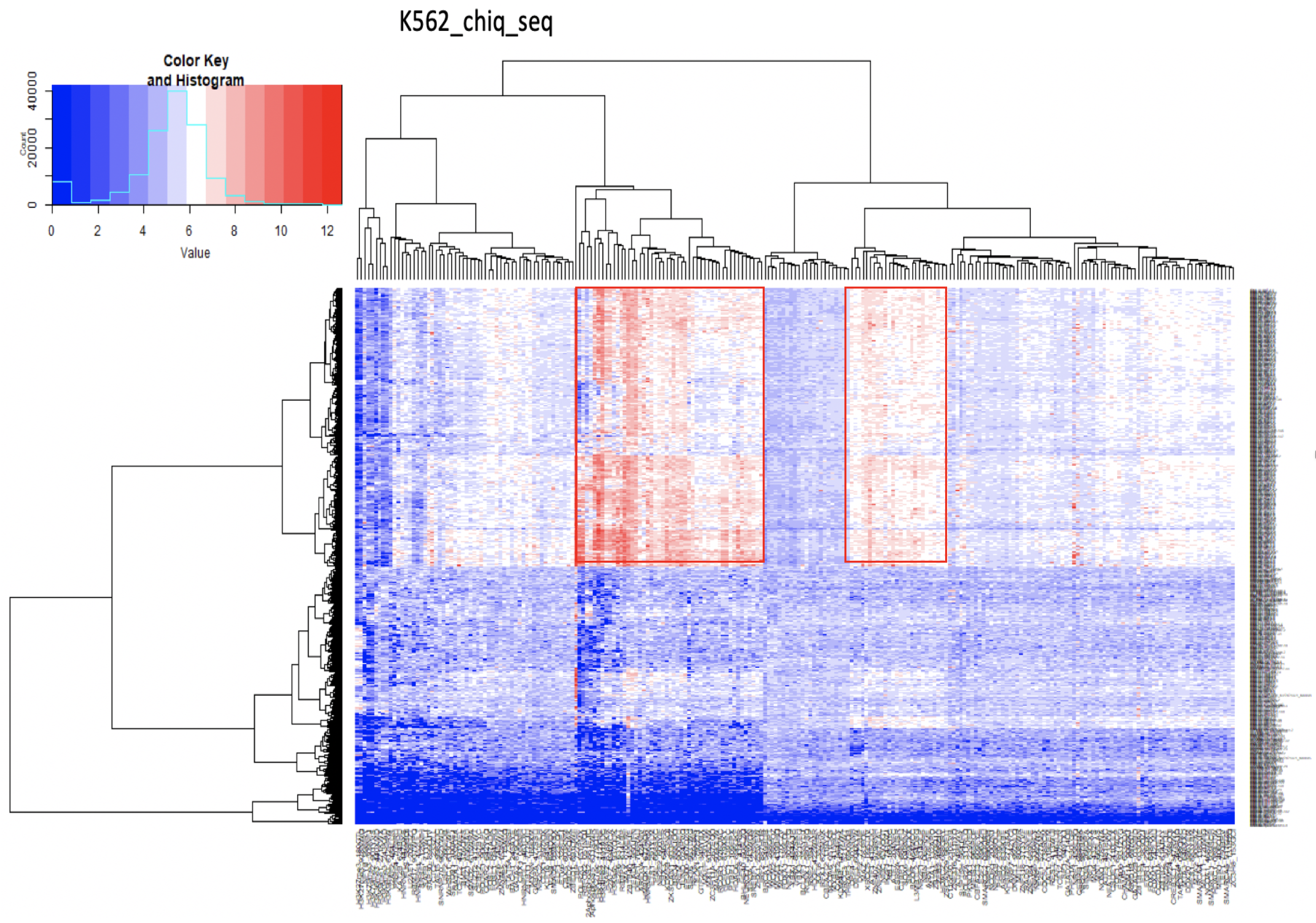


**Figure S2. The ChIP-Seq profiles of 243 DNA binding proteins (DBPs) and 10 histone modifications on tRNA genes in K562 cell.** The colored blocks in the matrix indicate binding intensities of DBPs or enrichment of histone modifications on the promoters of tRNA genes. The genes in red rectangles are considered as tRNA gene binding genes. The data can be found in the supplemental materials.

**Figure S3**


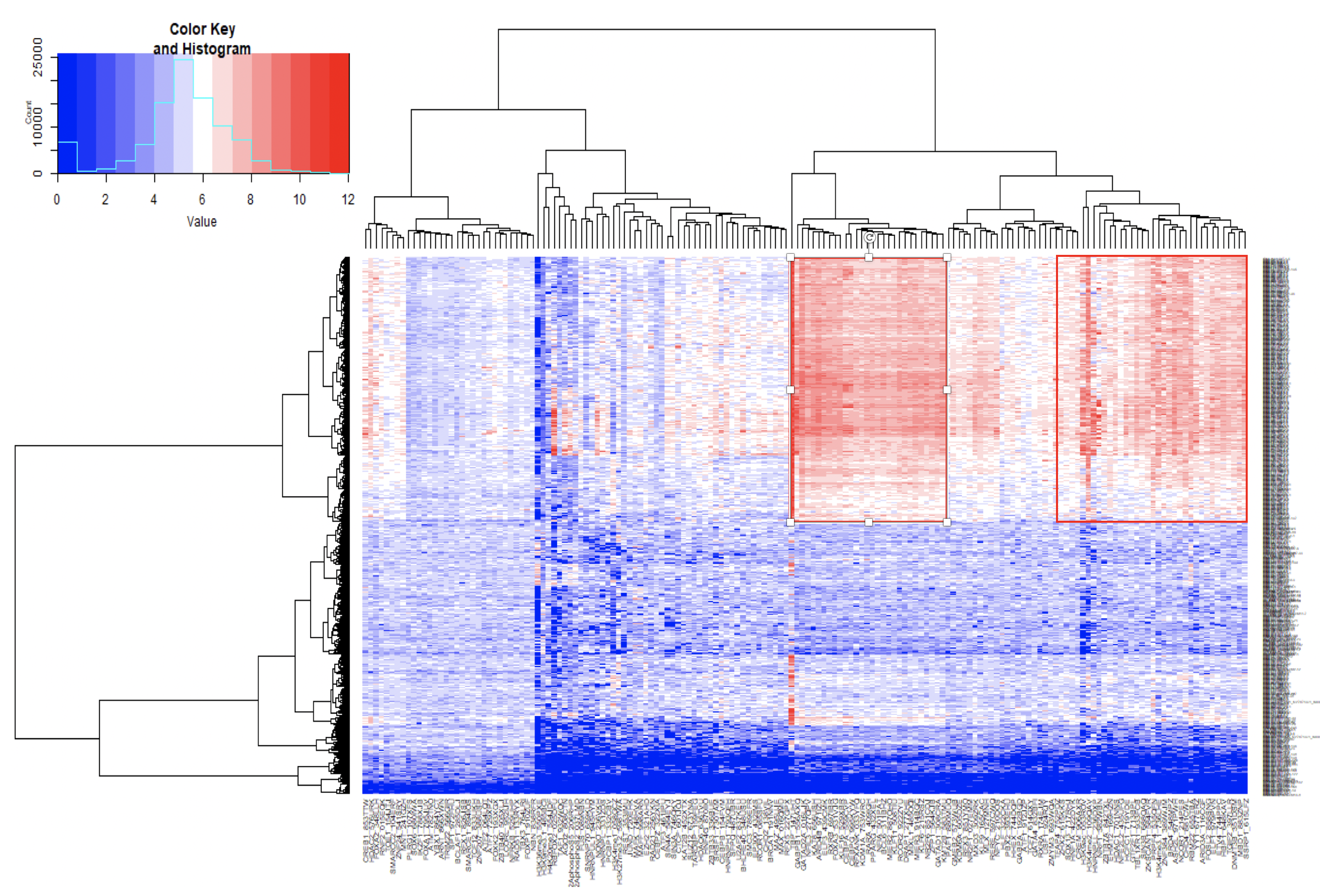


**Figure S3. The ChIP-Seq profiles of 155 DNA binding proteins (DBPs) and 9 histone modifications on tRNA genes in Hepg2 cell.** The colored blocks in the matrix indicate binding intensities of DBPs or enrichment of histone modifications on the promoters of tRNA genes. The genes in red rectangles are considered as tRNA gene binding genes. The data can be found in the supplemental materials.

**Figure S4**


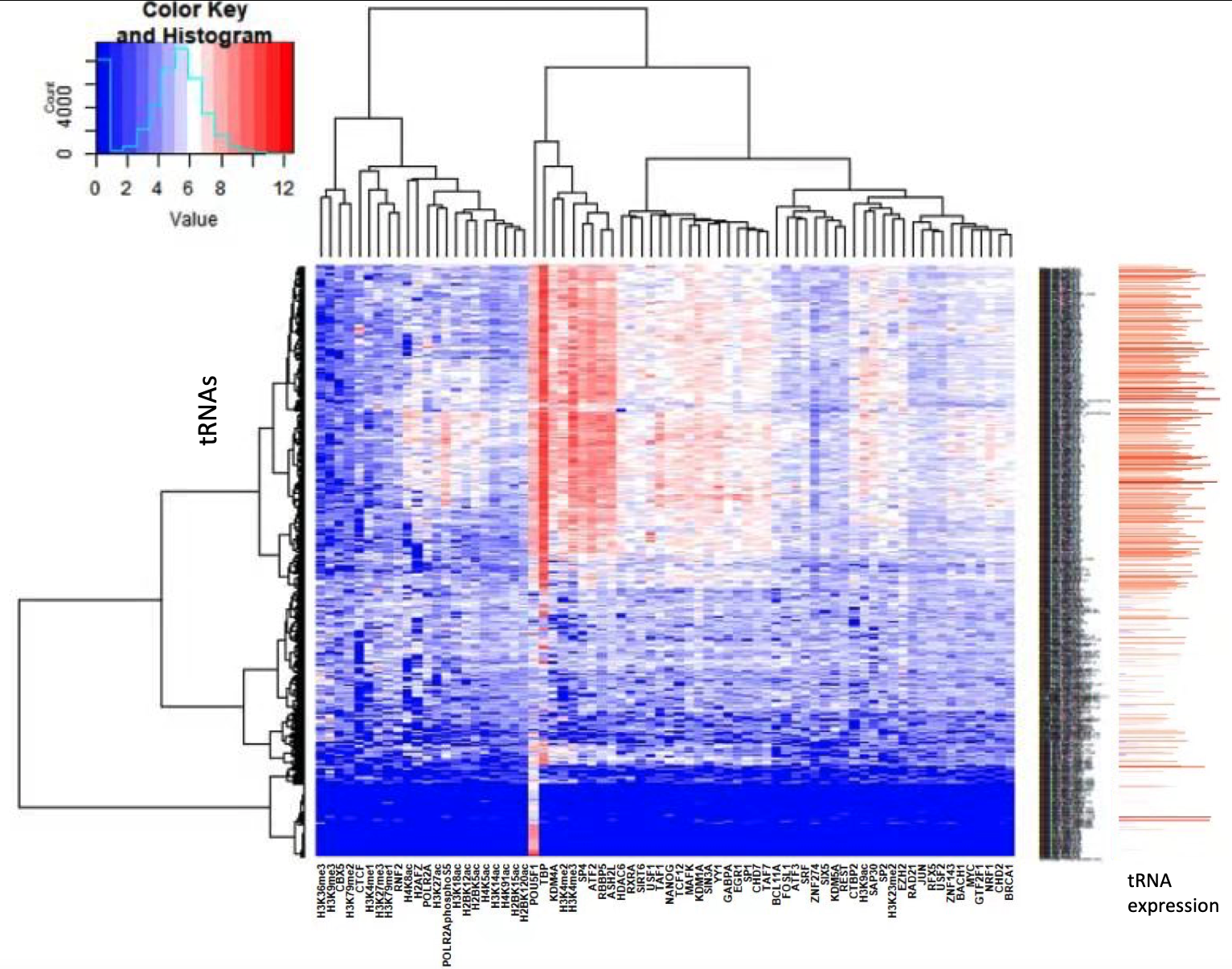


**Figure S4. ChIP-Seq profiles of 83 DNA binding proteins (DBPs) and 6 histone modifications on tRNA genes in H1-ES cell.** The colored blocks in the matrix indicate binding intensities of DBPs or enrichment of histone modifications on the promoters of tRNA genes. The bar chart on the right side indicates the abundance of tRNAs (LOG10(RPKM+1)). The genes in red rectangles are considered as tRNA gene binding genes.

**Figure S5**


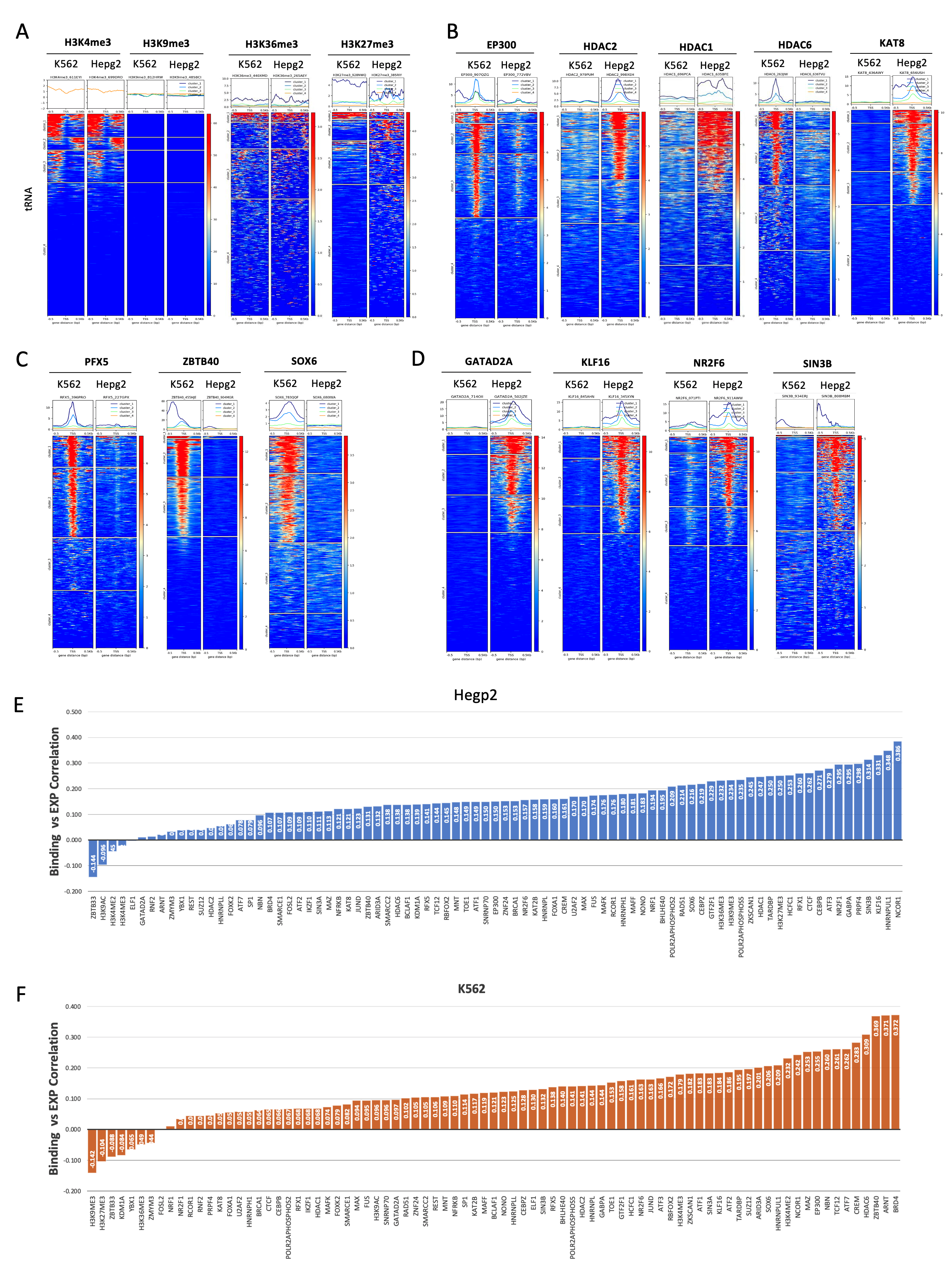


**Figure S5. Heatmaps of differential histone epigenetic markers and DBPs between K562 and Hepg2 cell lines.** A. The histone methylation profiles on tRNA genes (H3K4me3, H3K9me3, H3K36me3 and H3K27me3); B. The binding profiles of histone acetylation related enzymes (EP300, HDAC2, HDAC1, HDAC6 and KAT8) on tRNA genes; C. The tRNA gene binding profiles of transcriptional activators (PFX5, ZBTB40 and SOX6); D. The tRNA gene binding profiles of transcriptional repressors (GATAD2A, KLF16, NR2F6 and SIN3B); E, F. The Pearson correlation coefficients between the binding intensities of factors and tRNA gene expressions in the two cells.

**Figure S6**

**
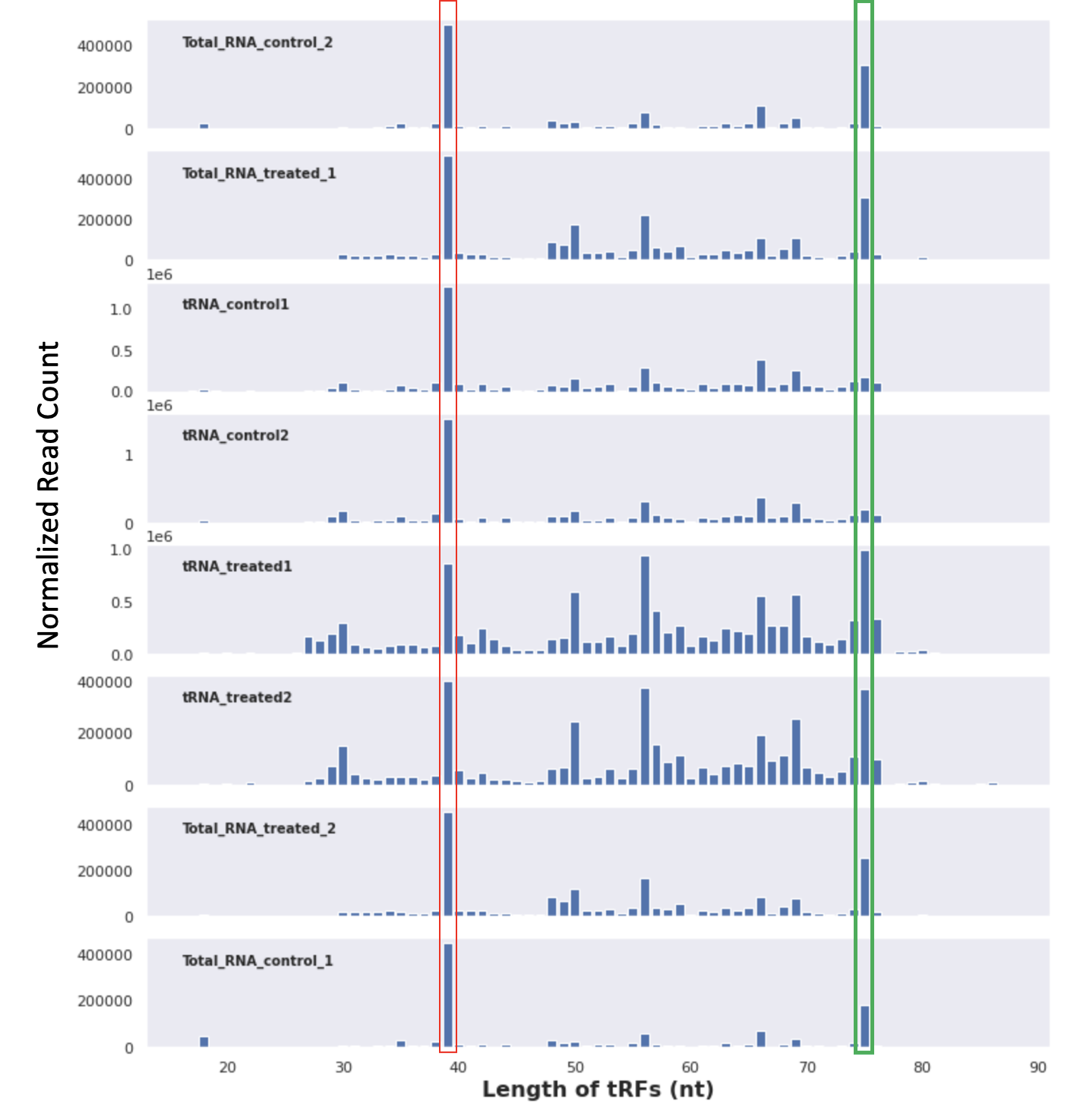
**

**Figure S6. The distribution of tRFs’ length in DM-tRNA-Seq data.** The read counts are normalized to total reads numbers. The red and green boxes indicate the peaks of tRNA-halves and full-length-tRNAs respectively.

**Figure S7.
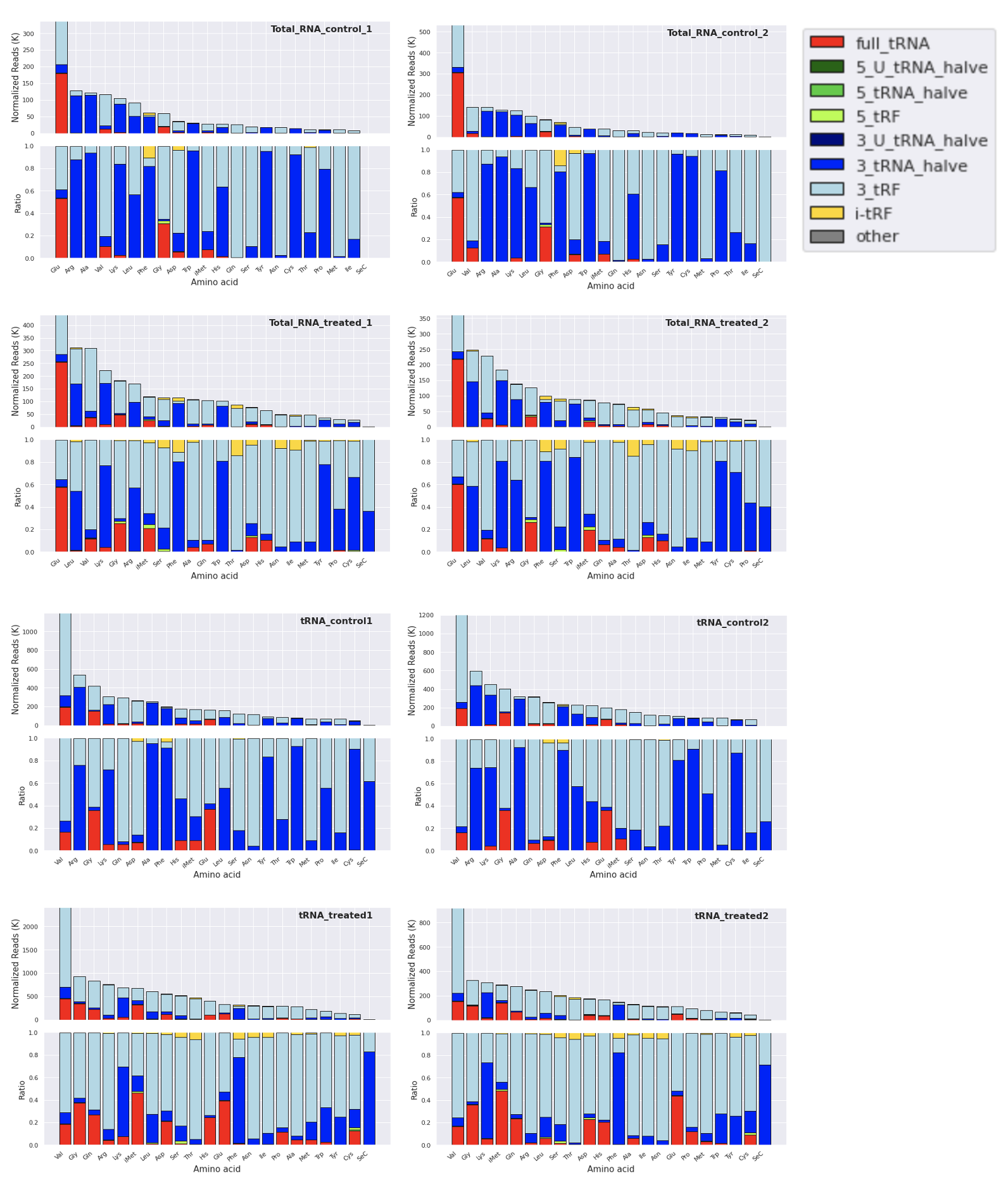
** **Figure S7. The tRF composition of tRNA isodecoders in DM-tRNA-Seq data.**

**Figure S8**


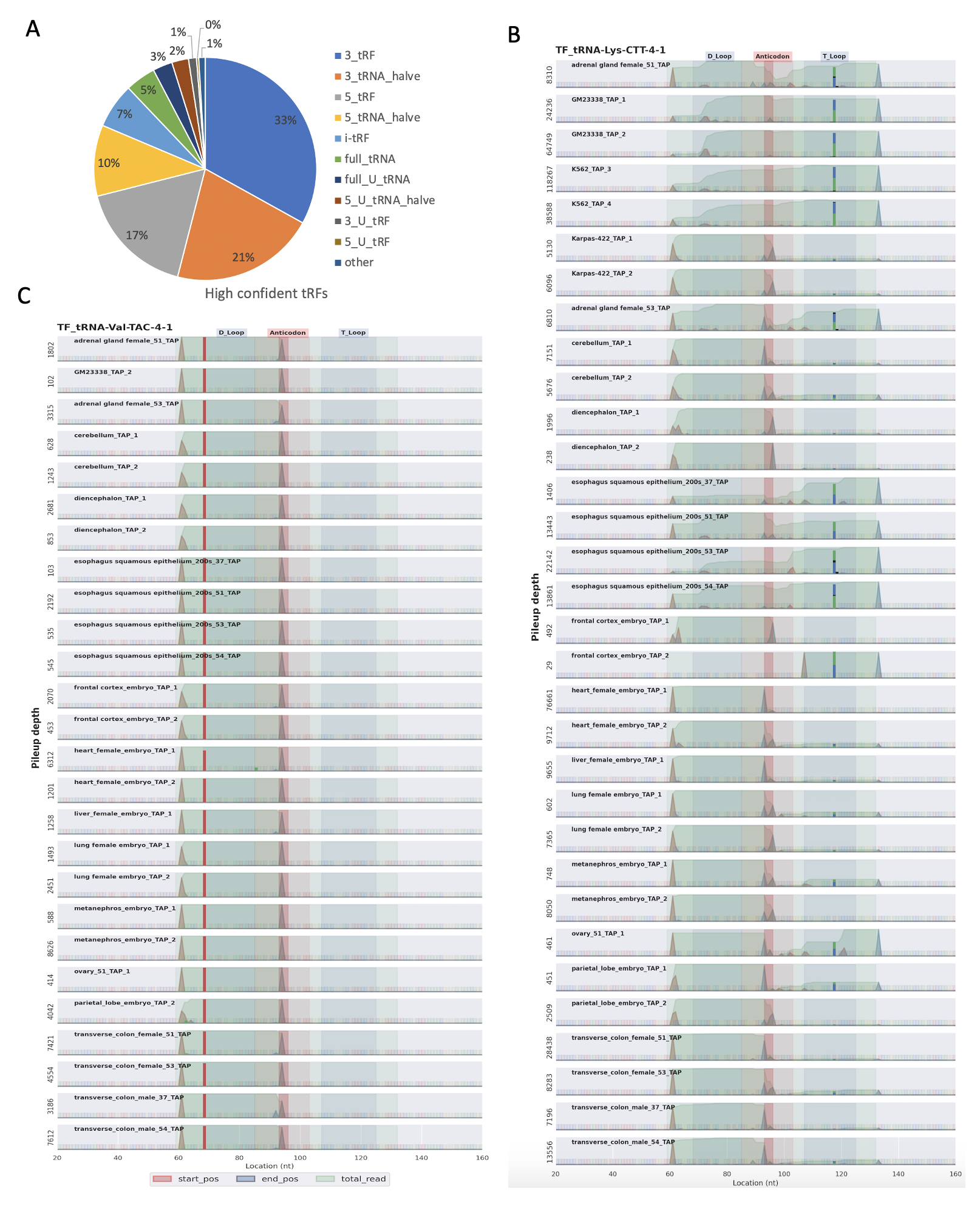


**Figure S8.** **tRFs distribution and tRF profiles in Cell Lines-Tissues data.** A. Pie chart to show the classification of high confident tRFs; B, C. the pileup profiles of TF-tRNA-Lys-CTT-4-1 (B) and TF_tRNA-Val-TAC-4-1(C). The red and blue peaks represent the start and end positions of tRFs. The light red and green regions represent the pileup reads from unique mapped reads and total mapped reads.

**Figure S9**


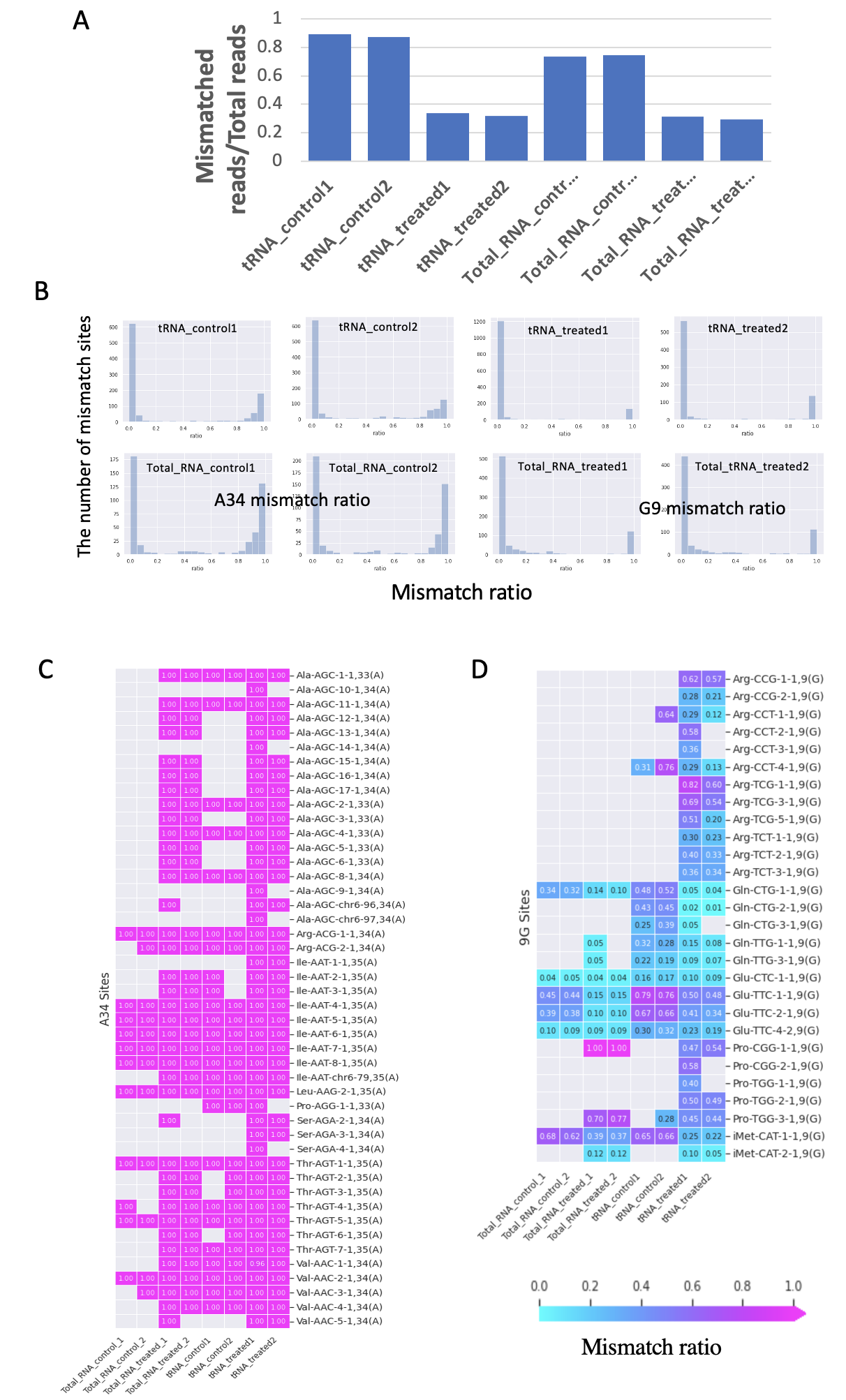


**Figure S9.** **The mismatches and modifications in DM-tRNA-Seq data.** A. The ratio of reads contains mismatches in DM-tRNA-Seq; B. The distribution of mismatch ratio in DM-tRNA-Seq data; C,D. A34 and G9 mismatch pattern in eight samples.

**Figure S10**

**
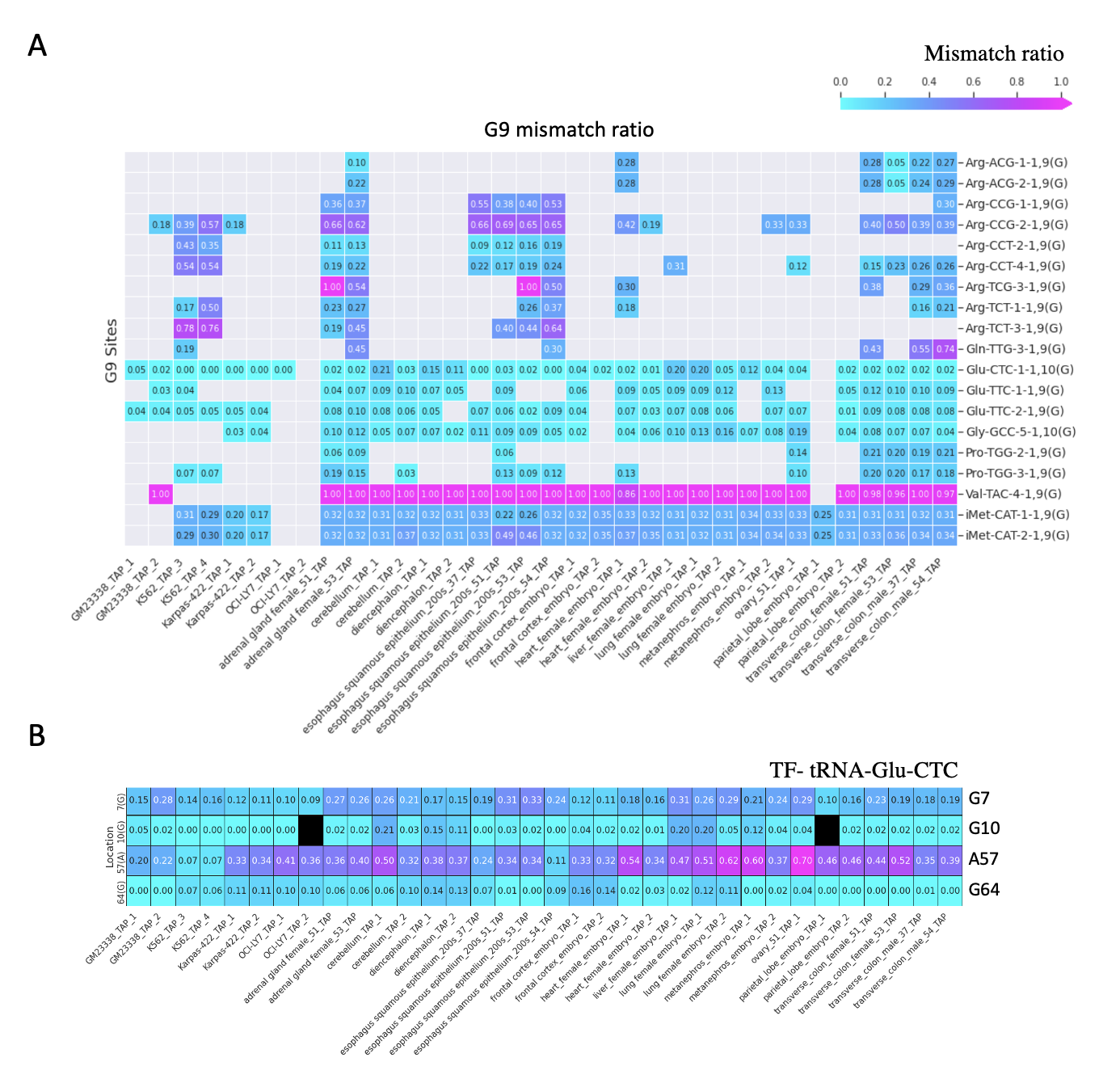
**

**Figure S10. The pattern of mismatches in Cell line and Tissue data.** A. Mismatch ratio of G9 of 19 tRNA families in 35 samples; B. Mismatch pattern of TF-tRNA-Glu-CTCs in 35 samples.

**Table S1: The hierarchical classification of tRNA genes and their fragments in human genome**

| Name | Level | Definition | Number |
| --- | --- | --- | --- |
| tRNA genome | 0 | The collection of all tRNA genes in genome | 1 |
| tRNA isoaccepter | 1 | tRNA gene groups carrying the same amino acid, with additional stop and end signals | 24 |
| tRNA isodecoder | 2 | tRNA gene groups containing the same anticodon | 59 |
| tRNA Gene Familiy | 3 | tRNA genes which have the same sequence of mature tRNAs | 407 |
| tRNA gene | 4 | tRNA gene annotated by GtRNAdb database | 619 |
| tRFs | 5 | Including 12 types of tRNA fragments | >4000 |

**Table S2. Top tRNA gene binding proteins/genes in HepG2**

| Gene | Function | Pathways |
| --- | --- | --- |
| HDAC1 | Histone deacetylases | Sin3 complex |
| NCOR1 | Mediates transcriptional repression by certain nuclear receptors, part of a complex | NCOR1-HDAC3 complex |
| GATAD2A | Transcriptional repressor, part of MBD2/NuRD nucleosome remodeling and deacetylase complex | HDACs deacetylate histones pathway |
| KLF16 | Transcriptional repressor, | KLF16-SIN3A complex |
| KAT8 | Histone acetyltransferase which may be involved in transcriptional activation | acetylation of nucleosomal histone H4 passway |
| GTF2F1 | Transcription initiation factor,it promotes transcription elongation | Formation of RNA Pol II elongation complex |
| PRPF4 | Involved in spliceosome assembly | mRNA Splicing - Major Pathway |
| NR2F1 | Represses transcriptional activity of LHCG | Nuclear Receptor transcription pathway |
| KDM1A | Histone demethylase that can demethylate both 'Lys-4' (H3K4me) and 'Lys-9' (H3K9me) of histone H3, thereby acting as a coactivator or a corepressor, depending on the context .Acts as a corepressor by mediating demethylation of H3K4me, a specific tag for epigenetic transcriptional activation. Demethylates both mono- (H3K4me1) and di-methylated (H3K4me2) H3K4me .Component of a RCOR/GFI/KDM1A/HDAC complex that suppresses, via histone deacetylase (HDAC) recruitment | HDACs deacetylate histones |
| NR2F6 | Transcriptional repressor | Nuclear Receptor transcription pathway |
| ELF1 | [Transcriptional activator](https://www.ebi.ac.uk/QuickGO/term/GO:0001228) | regulates transcription of genes involved in BCR signaling |
| HDAC2 | Responsible for the deacetylation of lysine residues on the N-terminal part of the core histones (H2A, H2B, H3 and H4) | Component of a RCOR/GFI/KDM1A/HDAC complex that suppresses, via histone deacetylase (HDAC) recruitment |
| CEBPB | Important transcription factor regulating the expression of genes involved in immune and inflammatory responses | Transcriptional regulation of white adipocyte differentiation |
| HCFC1 | Tethers the chromatin modifying Set1/Ash2 histone H3 'Lys-4' methyltransferase (H3K4me) and Sin3 histone deacetylase (HDAC) complexes (involved in the activation and repression of transcription, respectively) together | HATs acetylate histones |
| ZKSCAN1 | May be involved in transcriptional regulation | Generic Transcription Pathway |
| BRD4 | Chromatin reader protein that recognizes and binds acetylated histones and plays a key role in transmission of epigenetic memory across cell divisions and transcription regulation | BRD4-POLR2A complex |
| SIN3B | Transcriptional repressor | forms a complex with FOXK1 which represses transcription |
| FOSL2 | As a dimer with JUN, activates LIF transcription. Activates CEBPB transcription in PGE2-activated osteoblasts | FOSL2-JUN complex |
| ARID3A | [Transcriptional activator](https://www.ebi.ac.uk/QuickGO/term/GO:0001228) | TP53 Regulates Transcription of Genes Involved in G1 Cell Cycle Arrest |
| CREM | Transcriptional regulator that binds the cAMP response element (CRE). Isoforms are either transcriptional activators or repressors | FHL5-CREM complex |

**Table S3. Top tRNA gene binding proteins/genes in K562**

| Gene | Function | Pathways |
| --- | --- | --- |
| ZBTB40 | May be involved in transcriptional regulation | - |
| IKZF1 | Regulates transcription through association with both HDAC-dependent and HDAC-independent complexes | NOTCH3 Intracellular Domain Regulates Transcription |
| MAZ | May function as a transcription factor with dual roles in transcription initiation and termination | MAPK3-MAZ-CSNK2A1 complex |
| NBN | Playing a critical role in the cellular response to DNA damage and the maintenance of chromosome integrity | MRE11-RAD50-NBN complex |
| ARNT | Transcriptional activator | Aryl hydrocarbon receptor signalling |
| NR2F | Transcription factor predominantly involved in transcriptional repression | Nuclear Receptor transcription pathway |
| ATF7 | Binds the cAMP response element (CRE) (consensus: 5'-GTGACGT[AG][AG]-3'), a sequence present in many viral and cellular promoters. Usually activate transcription. However, some isoforms can acts as a negative regulator. | TAF4-ATF7-TAF12 |
| ATF1 | Transcriptional activator | CREB phosphorylation |
| REST | Transcriptional repressor, restricts the expression of neuronal genes by associating with two distinct corepressors, SIN3A and RCOR1, which in turn recruit histone deacetylase to the promoters of REST-regulated genes | REST-SIN3A-RCOR1 complex |
| HDAC6 | Responsible for the deacetylation of lysine residues on the N-terminal part of the core histones (H2A, H2B, H3 and H4).  Involved in the MTA1-mediated epigenetic regulation of ESR1 expression in breast cancer. | HDAC6-SRSF2 complex(deacetylation)\  MAPK3-HDAC6 complex(phosphorylation) |
| JUND | Transcriptional activator | JUND-MAPK3-MAPK8-MAPK1 complex |
| HNRNPL | Splicing factor binding to exonic or intronic sites and acting as either an activator or repressor of exon inclusion | HNRNPL-APEX1 complex |
| MAX | The MYC:MAX complex is a transcriptional activator, whereas the MAD:MAX complex is a repressor.  May repress transcription via the recruitment of a chromatin remodeling complex containing H3 'Lys-9' histone methyltransferase activity | Transcriptional Regulation by E2F6 |
| BHLHE40 | Transcriptional repressor | BMAL1:CLOCK,NPAS2 activates circadian gene expression |
| CREM | Transcriptional regulator that binds the cAMP response element (CRE). Isoforms are either transcriptional activators or repressors | FHL5-CREM complex |
| ZKSCAN1 | May be involved in transcriptional regulation | Generic Transcription Pathway |
| RCOR1 | Essential component of the BHC complex, a corepressor complex that represses transcription of neuron-specific genes in non-neuronal cells. The BHC complex is recruited at RE1/NRSE sites by REST and acts by deacetylating and demethylating specific sites on histones, thereby acting as a chromatin modifier. | HDACs deacetylate histones |
| NR2F6 | Transcriptional repressor | Nuclear Receptor transcription pathway |
| ARID3A | [Transcriptional activator](https://www.ebi.ac.uk/QuickGO/term/GO:0001228) | TP53 Regulates Transcription of Genes Involved in G1 Cell Cycle Arrest |
| HCFC1 | Tethers the chromatin modifying Set1/Ash2 histone H3 'Lys-4' methyltransferase (H3K4me) and Sin3 histone deacetylase (HDAC) complexes (involved in the activation and repression of transcription, respectively) together | HATs acetylate histones |
| SIN3A | Acts cooperatively with OGT to repress transcription in parallel with histone deacetylation | SIN3A-MXI1 complex(repress MYC responsive genes) |
| EP300 | Functions as histone acetyltransferase and regulates transcription via chromatin remodeling | HATs(histone acetyltransferase) acetylate histones |
| BRD4 | Chromatin reader protein that recognizes and binds acetylated histones and plays a key role in transmission of epigenetic memory across cell divisions and transcription regulation | BRD4-POLR2A complex |

**Table S4. Top tRNA gene binding genes in H1-ES cell line**

| Gene | Function | Pathways |
| --- | --- | --- |
| MAFK | transcriptional repressors(dimerize among themselves)/transcriptional activators(NFE2, NFE2L1/NRF1, NFE2L2/NRF2 and NFE2L3/NRF3) | Factors involved in megakaryocyte development and platelet production |
| ASH2L | Component of the Set1/Ash2 histone methyltransferase (HMT) complex, a complex that specifically methylates 'Lys-4' of histone H3, but not if the neighboring 'Lys-9' residue is already methylated. As part of the MLL1/MLL complex it is involved in methylation and dimethylation at 'Lys-4' of histone H3 | SET1-ASH2L complex/  ASH2L-MLL1 complex |
| RBBP5 | In embryonic stem (ES) cells,regulating gene induction and H3 'Lys-4' methylation at key developmental loci.As part of the MLL1/MLL complex, involved in mono-, di- and trimethylation at 'Lys-4' of histone H3. | RBBP5-MLL/SET |
| SP4 | [transcription coactivator](https://www.ebi.ac.uk/QuickGO/term/GO:0003713) | SP4 up-regulates quantity by expression to MAOB |
| ATF2 | Exhibits histone acetyltransferase (HAT) activity which specifically acetylates histones H2B and H4 in vitro. Can elicit oncogenic or tumor suppressor activities depending on the tissue or cell type | HATs acetylate histones |
| SP2 | [DNA-binding transcription repressor activity](https://www.ebi.ac.uk/QuickGO/term/GO:0001227).  [histone deacetylase binding](https://www.ebi.ac.uk/QuickGO/term/GO:0042826) | SP2 up-regulates activity to IRF1 |
| HDAC6 | Responsible for the deacetylation of lysine residues on the N-terminal part of the core histones (H2A, H2B, H3 and H4). Involved in the MTA1-mediated epigenetic regulation of ESR1 expression in breast cancer. | HDAC6-SRSF2 complex(deacetylation)\  MAPK3-HDAC6 complex(phosphorylation) |
| KDM1A | Histone demethylase that can demethylate both 'Lys-4' (H3K4me) and 'Lys-9' (H3K9me) of histone H3, thereby acting as a coactivator or a corepressor, depending on the context .   Acts as a corepressor by mediating demethylation of H3K4me, a specific tag for epigenetic transcriptional activation. Demethylates both mono- (H3K4me1) and di-methylated (H3K4me2) H3K4me .  Component of a RCOR/GFI/KDM1A/HDAC complex that suppresses, via histone deacetylase (HDAC) recruitment | HDACs deacetylate histones (RCOR/GFI/KDM1A/HDAC complex) |
| POU5F1 | Also named OCT4, key transcription factor for maintaining pluripotent of stem cells. | Pluripotent stem cell pathway |
| KDM4A | Histone demethylase that specifically demethylates 'Lys-9' and 'Lys-36' residues of histone H3, thereby playing a central role in histone code. Does not demethylate histone H3 'Lys-4', H3 'Lys-27' nor H4 'Lys-20'. Demethylates trimethylated H3 'Lys-9' and H3 'Lys-36' residue, while it has no activity on mono- and dimethylated residues. Demethylation of Lys residue generates formaldehyde and succinate. | HDMs (Histone lysine demethylases) demethylate histones |
| NRF1 | Transcription factor that activates the expression of the EIF2S1 (EIF2-alpha) gene | Transcriptional activation of mitochondrial biogenesis |
| SIN3A | Acts cooperatively with OGT to repress transcription in parallel with histone deacetylation | SIN3A-MXI1 complex(repress MYC responsive genes) |

**Table S5. Mismatch sites identified in the DM-tRNA-Seq data**

| Isoacceptor | members | 33A | 36A | 58A | 52A | 67A | 69A | 25G | 36G | 56G | 32C | 51C | 35U | 27U | 28U | 25C |  |
| --- | --- | --- | --- | --- | --- | --- | --- | --- | --- | --- | --- | --- | --- | --- | --- | --- | --- |
| tRFA-Ala (AGC) | **15** | **+** | **+** | **+** | **-** | **-** | **-** | **-** | **-** | **-** | **-** | **-** | **-** | **-** | **-** | **+** |  |
| tRFA-Ala (CGC) | **4** | **-** | **+** | **+(-1)** | **-** | **-** | **-** | **+** | **-** | **-** | **-** | **-** | **-** | **-** | **-** | **-** |  |
| tRFA-Ala (TGC) | **6** | **-** | **+** | **+(-1)** | **-** | **-** | **-** | **+(+1)** | **-** | **+** | **-** | **-** | **-** | **-** | **-** | **-** |  |
| tRNA-Arg (ACG) | **2** | **+** | **-** | **+** | **-** | **-** | **-** | **+** | **-** | **-** | **-** | **-** | **-** | **-** | **-** | **-** |  |
| tRNA-Arg (CCG) | **2** | **-** | **-** | **+** | **-** | **-** | **-** | **+** | **-** | **-** | **-** | **-** | **-** | **-** | **-** | **-** |  |
| tRNA-Arg (CCT) | **4** | **-** | **-** | **+** | **-** | **-** | **-** | **-** | **-** | **-** | **-** | **-** | **-** | **-** | **-** | **-** |  |
| tRNA-Arg (TCG) | **5** | **-** | **-** | **+** | **-** | **-** | **-** | **+** | **-** | **-** | **-** | **-** | **-** | **-** | **-** | **-** |  |
| tRNA-Arg (TCT)-4 | **1** | **-** | **-** | **+(+1)** | **-** | **-** | **-** | **-** | **-** | **-** | **-** | **-** | **-** | **-** | **-** | **-** |  |
| tRNA-Arg (TCT)-1~3 | **3** | **-** | **+(+1)** | **+(+1)** | **-** | **-** | **-** | **-** | **-** | **-** | **-** | **-** | **-** | **-** |  |  |  |
| tRNA-Asn (GTT) | **10** | **-** | **-** | **+(+1)** | **-** | **-** | **-** | **+** | **-** | **-** | **-** | **-** | **-** | **-** | **-** | **-** |  |
| tRNA-Cys (GCA) | **12** | **-** | **-** | **+(-1)** | **-** | **-** | **-** | **-** | **-** | **-** | **-** | **-** | **-** | **-** | **-** | **-** |  |
| tRNA-Gln (CTG) | **6** | **-** | **-** | **+(-1)** | **-** | **-** | **-** | **-** | **-** | **-** | **-** | **-** | **-** | **-** | **-** | **-** |  |
| tRNA-Gln (TTG) | **6** | **-** | **-** | **+(-1)** | **-** | **-** | **-** | **-** | **-** | **-** | **-** | **-** | **-** | **-** | **-** | **-** |  |
| tRNA-Glu (TTC) | **1/4** | **-** | **-** | **+** | **-** | **-** | **-** | **-** | **-** | **-** | **-** | **-** | **-** | **-** | **-** | **-** |  |
| tRNA-Gly (CCC) | **2** | **-** | **-** | **+** | **-** | **-** | **-** | **-** | **-** | **-** | **-** | **-** | **-** | **-** | **-** | **-** |  |
| tRNA-Gly (GCC) | **2** | **-** | **-** | **+(-2)** | **-** | **-** | **-** | **-** | **-** | **-** | **-** | **-** | **-** | **-** | **-** | **-** |  |
| tRNA-Gly (TCC) | **2** | **-** | **-** | **+(-1)** | **-** | **-** | **-** | **-** | **-** | **-** | **-** | **-** | **-** | **-** | **-** | **-** |  |
| tRNA-His (GTG) | **1** | **-** | **-** | **+** | **-** | **-** | **-** | **-** | **-** | **-** | **-** | **-** | **-** | **-** | **-** | **-** |  |
| tRNA-Ile (AAT) | **9** | **+** | **-** | **+(+1)** | **-** | **-** | **-** | **+** | **-** | **-** | **-** | **-** | **-** | **-** | **-** | **-** |  |
| tRNA-Ile (TAT) | **3** | **-** | **+(+1)** | **-** | **-** | **-** | **-** | **+** | **-** | **-** | **-** | **-** | **-** | **-** | **-** | **-** |  |
| tRNA-iMet (CAT) | **1** | **-** | **-** | **+(-1)** | **-** | **-** | **-** | **-** | **-** | **-** | **-** | **-** | **-** | **-** | **-** | **-** |  |
| tRNA-Leu (AAG) | **2** | **-** | **+(-1)** | **+(+9i)** | **-** | **-** | **-** | **-** | **-** | **-** | **-** | **-** | **-** | **-** | **-** | **-** |  |
| tRNA-Leu (CAA) | **4** | **-** | **-** | **-** | **-** | **-** | **-** | **+** | **-** | **-** | **-** | **-** | **-** | **-** | **-** | **-** |  |
| tRNA-Leu (CAG) | **2** | **-** | **-** | **+(*)** | **-** | **-** | **-** | **+** | **-** | **-** | **-** | **+(*)** | **-** | **-** | **-** | **-** |  |
| tRNA-Leu (TAG) | **3** | **-** | **-** | **+(*)** | **-** | **-** | **-** | **-** | **-** | **-** | **-** | **-** | **+** | **-** | **-** | **-** |  |
| tRNA-Lys (CTT) | **4** | **-** | **-** | **+** | **-** | **-** | **-** | **-** | **-** | **-** | **-** | **-** | **-** | **-** | **-** | **-** |  |
| tRNA-Lys (TTT) | **14** | **-** | **-** | **+** | **+** | **-** | **+** | **-** | **-** | **-** | **-** | **-** | **-** | **-** | **-** | **-** |  |
| tRNA-Met (CAT) | **6** | **-** | **-** | **+** | **-** | **-** | **-** | **-** | **-** | **-** | **-** | **-** | **-** | **-** | **-** | **-** |  |
| tRNA-Phe (GAA) | **6** | **-** | **-** | **+** | **-** | **-** | **-** | **+(+1)** | **-** | **-** | **-** | **-** | **-** | **+** | **+** | **+** |  |
| tRNA-Pro (CGG) | **2** | **-** | **-** | **+(-1)** | **-** | **-** | **-** | **-** | **+** | **-** | **-** | **-** | **-** | **-** | **-** | **-** |  |
| tRNA-Pro (TGG) | **3** | **-** | **-** | **-** | **-** | **-** | **-** | **-** | **+** | **-** | **-** | **-** | **-** | **-** | **-** | **-** |  |
| tRNA-SeC (TCA) | **1** | **-** | **-** | **+(+13)** | **-** | **-** | **-** | **-** | **-** | **-** | **-** | **-** | **-** | **-** | **-** | **-** |  |
| tRNA-Ser (AGA) | **5** | **+(+1)** | **-** | **+(+9)** | **-** | **-** | **-** | **+(+1)** | **-** | **-** | **-** | **-** | **-** | **-** | **-** | **-** |  |
| tRNA-Ser (CGA) | **4** | **-** | **-** | **+(+9)** | **-** | **-** | **-** | **+(+1)** | **-** | **-** | **-** | **-** | **-** | **-** | **-** | **-** |  |
| tRNA-Ser (GCT) | **6** | **-** | **-** | **+(+9)** | **-** | **-** | **-** | **+(+1)** | **-** | **-** | **-** | **+(+1)** | **-** | **-** | **-** | **-** |  |
| tRNA-Ser (TGA) | **4** | **-** | **-** | **+(+9)** | **-** | **-** | **-** | **+(+1)** | **-** | **-** | **+** | **-** | **-** | **-** | **-** | **-** |  |
| tRNA-Thr (AGT) | **7** | **+(+2)** | **-** | **+(+1)** | **-** | **-** | **-** | **+(+2)** | **-** | **-** | **-** | **-** | **-** | **-** | **-** | **-** |  |
| tRNA-Thr (CGT) | **4** | **+(+2)** | **-** | **+(+1)** | **-** | **-** | **-** | **+(+2)** | **-** | **-** | **-** | **-** | **-** | **-** | **-** | **-** |  |
| tRNA-Thr (TGT) | **6** | **+(-1, +1)** | **-** | **+** | **-** | **-** | **-** | **+(+2)** | **-** | **-** | **-** | **-** | **-** | **-** | **-** | **-** |  |
| tRNA-Trp (CCA) | **5** | **-** | **-** | **+(+1)** | **-** | **-** | **-** | **+** | **-** | **-** | **-** | **-** | **-** | **-** | **-** | **-** |  |
| tRNA-Tyr (GTA) | **8** | **-** | **-** | **+** | **-** | **-** | **-** | **-** | **+(+1)** | **-** | **-** | **-** | **-** | **-** | **-** | **-** |  |
| tRNA-Val (AAC) | **5** | **+(+1)** | **-** | **+** | **-** | **+** | **-** | **-** | **-** | **-** | **-** | **-** | **-** | **-** | **-** | **-** |  |
| tRNA-Val (CAC) | **4** | **-** | **-** | **+** | **-** | **-** | **-** | **-** | **-** | **-** | **-** | **-** | **-** | **-** | **-** | **-** |  |
| tRNA-Val (TAC) | **2/4** | **-** | **-** | **+** | **-** | **-** | **-** | **-** | **-** | **-** | **-** | **-** | **-** | **-** | **-** | **-** |  |
| Notice: + means existed, - means didn't found, (+n) means the location shift to right for n bases. For example, tRNA-Asn (GTT) has a 58A (+1) mutations which means the location of 58A actually is the 59th position. | | | | | | | | | | | | | | | | | |
