# Supplementary material for: Integrated genomic analysis reveals regulatory pathways and dynamic landscapes of the tRNA transcriptome
Source: Sci Rep. 2021 Mar 4;11:5226. doi: 10.1038/s41598-021-83469-6 (PMC7933247; doi:10.1038/s41598-021-83469-6)
Supplement: Supplementary file 2 — Supplementary Data. [file 41598_2021_83469_MOESM2_ESM.zip › 5_DREME_Motif.pdf]

DREME
Discriminative Regular Expression Motif Elicitation

For further information on how to interpret these results please access http://meme-suite.org/.
To get a copy of the MEME software please access http://meme-suite.org.

If you use DREME in your research please cite the following paper:
Timothy L. Bailey, "DREME: Motif discovery in transcription factor ChIP-seq data", *Bioinformatics*, 27(12):1653-1659, 2011. [full text]

DISCOVERED MOTIFS | INPUTS & SETTINGS | PROGRAM INFORMATION | RESULTS IN TEXT FORMAT NEW! | RESULTS IN XML FORMAT NEW!

DISCOVERED MOTIFS

Next Top

	Motif	Logo	RC Logo	E-value	Unerased E-value	More	Submit/Dov
1.	GTTCRA	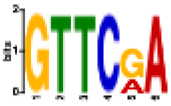	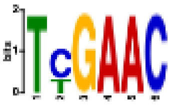	6.3e-279	6.3e-279	↥	⇢

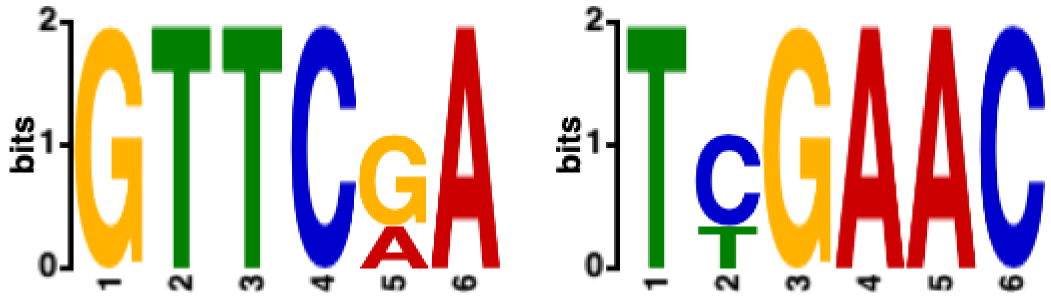

Details

Positives	Negatives	P-value	E-value	Unerased E-value
1757 / 19952	264 / 19952	3.3e-283	6.3e-279	6.3e-279

Enriched Matching Words

Word	Positives	Negatives	P-value	E-value
GTTCGA	1199 / 19952	108 / 19952	2.3e-240	4.5e-236
GTTCAA	558 / 19952	156 / 19952	4.6e-055	8.8e-051

	Motif	Logo	RC Logo	E-value	Unerased E-value	More	Submit/Dov
2.	CAGATYA	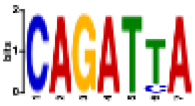	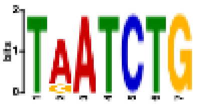	4.4e-091	4.4e-091	↥	⇢

	Motif	Logo	RC Logo	E-value	Unerased E-value	More	Submit/Dov

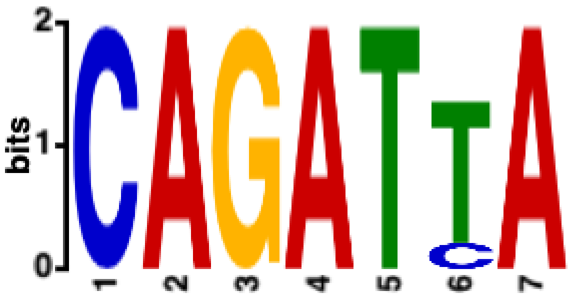

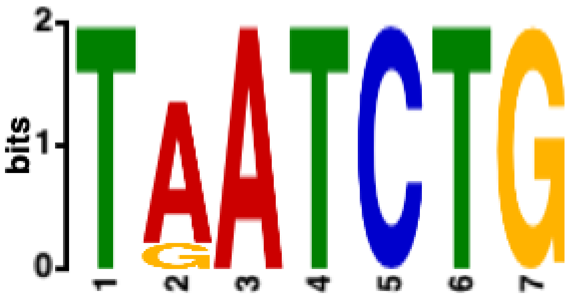

Details

Positives	Negatives	P-value	E-value	Unerased E-value
422 / 19952	25 / 19952	2.3e-095	4.4e-091	4.4e-091

Enriched Matching Words

Word	Positives	Negatives	P-value	E-value
CAGATTA	357 / 19952	12 / 19952	2.1e-090	4.0e-086
CAGATCA	65 / 19952	13 / 19952	8.7e-010	1.7e-005

	Motif	Logo	RC Logo	E-value	Unerased E-value	More	Submit/Dov
3.	CASRCG	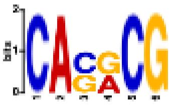	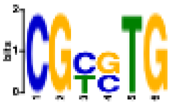	1.9e-077	3.9e-077	↥	⇢

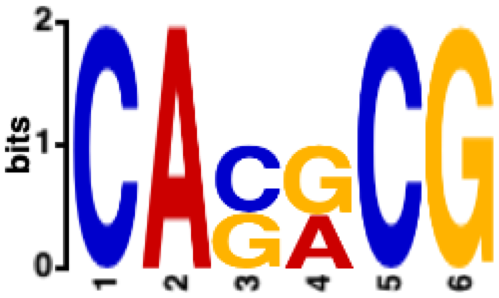

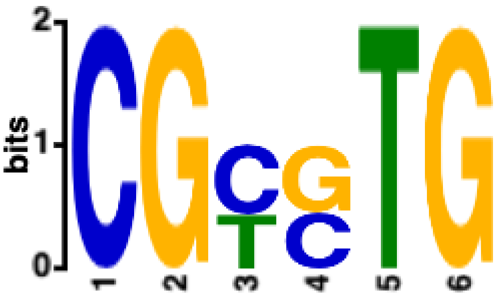

Details

Positives	Negatives	P-value	E-value	Unerased E-value
1010 / 19952	335 / 19952	1.0e-081	1.9e-077	3.9e-077

Enriched Matching Words

Word	Positives	Negatives	P-value	E-value
CACGCG	384 / 19952	98 / 19952	8.7e-042	1.6e-037
CACACG	277 / 19952	80 / 19952	4.6e-027	8.6e-023
CAGGCG	283 / 19952	85 / 19952	1.7e-026	3.2e-022
CAGACG	189 / 19952	80 / 19952	1.1e-011	2.0e-007

	Motif	Logo	RC Logo	E-value	Unerased E-value	More	Submit/Dov
4.	AGGCGAR	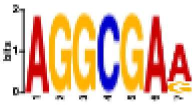	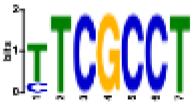	3.7e-056	6.7e-076	↥	⇢

Motif	Logo	RC Logo	E-value	Unerased E-value	More	Submit/Dov

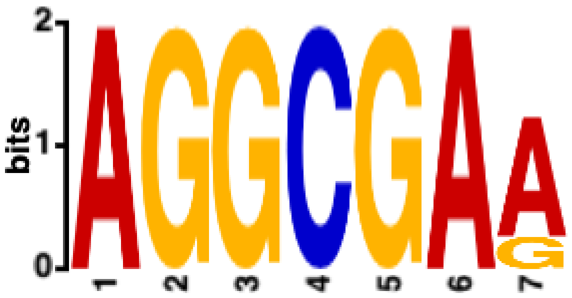
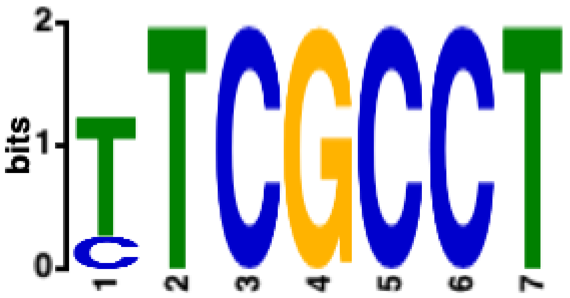

Details

Positives	Negatives	P-value	E-value	Unerased E-value
286 / 19952	22 / 19952	2.0e-060	3.7e-056	6.7e-076

Enriched Matching Words

Word	Positives	Negatives	P-value	E-value
AGGCGAA	225 / 19952	10 / 19952	1.2e-054	2.3e-050
AGGCGAG	61 / 19952	12 / 19952	2.3e-009	4.3e-005

	Motif	Logo	RC Logo	E-value	Unerased E-value	More	Submit/Dov
**5.**	GTTWWCA	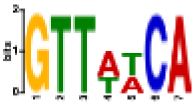	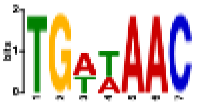	1.1e-047	3.5e-047	↥	⇢

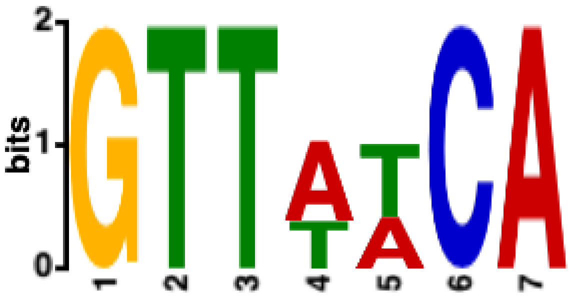
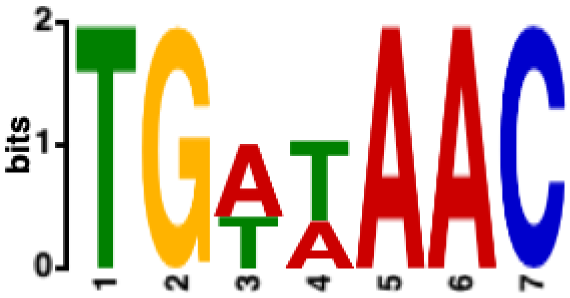

Details

Positives	Negatives	P-value	E-value	Unerased E-value
380 / 19952	73 / 19952	6.1e-052	1.1e-047	3.5e-047

Enriched Matching Words

Word	Positives	Negatives	P-value	E-value
GTTATCA	122 / 19952	11 / 19952	3.3e-025	6.0e-021
GTTAACA	116 / 19952	11 / 19952	1.3e-023	2.3e-019
GTTTTCA	99 / 19952	32 / 19952	1.7e-009	3.2e-005
GTTTACA	43 / 19952	19 / 19952	1.6e-003	2.9e+001

	Motif	Logo	RC Logo	E-value	Unerased E-value	More	Submit/Dov
**6.**	TAGCTCR	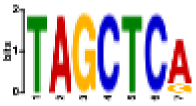	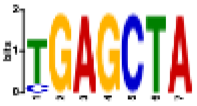	5.5e-047	5.5e-047	↥	⇢

	Motif	Logo	RC Logo	E-value	Unerased E-value	More	Submit/Dov

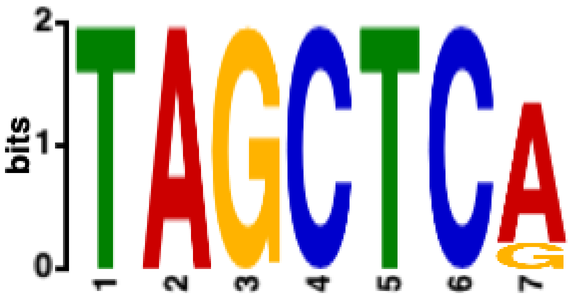
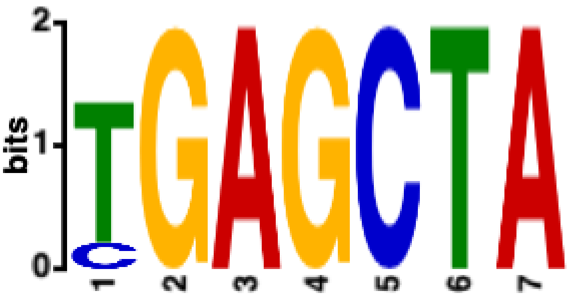

Details

Positives	Negatives	P-value	E-value	Unerased E-value
280 / 19952	32 / 19952	3.0e-051	5.5e-047	5.5e-047

Enriched Matching Words

Word	Positives	Negatives	P-value	E-value
TAGCTCA	236 / 19952	18 / 19952	3.4e-050	6.2e-046
TAGCTCG	44 / 19952	14 / 19952	5.0e-005	9.1e-001

	Motif	Logo	RC Logo	E-value	Unerased E-value	More	Submit/Dov
7.	GCTCTACC	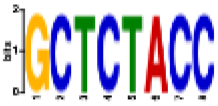	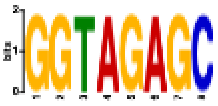	2.3e-044	2.3e-044	↥	⇢

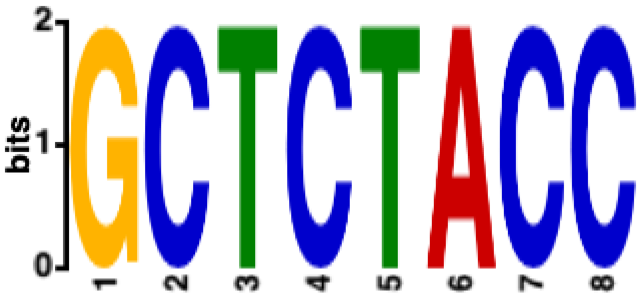
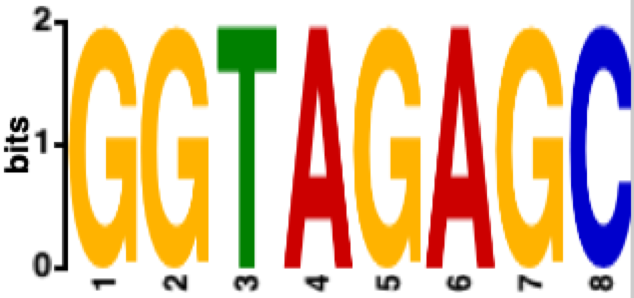

Details

Positives	Negatives	P-value	E-value	Unerased E-value
165 / 19952	1 / 19952	1.3e-048	2.3e-044	2.3e-044

Enriched Matching Words

Word	Positives	Negatives	P-value	E-value
GCTCTACC	165 / 19952	1 / 19952	1.3e-048	2.3e-044

	Motif	Logo	RC Logo	E-value	Unerased E-value	More	Submit/Dov
8.	CTCNTAA	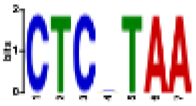	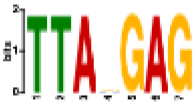	3.0e-042	4.6e-048	↥	⇢

Motif	Logo	RC Logo	E-value	Unerased E-value	More	Submit/Dov

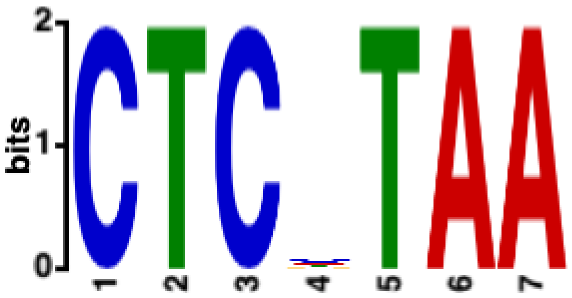

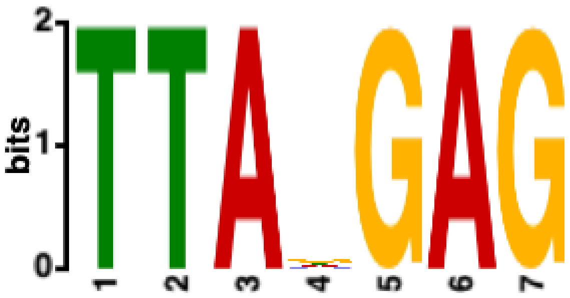

Details

Positives	Negatives	P-value	E-value	Unerased E-value
310 / 19952	52 / 19952	1.6e-046	3.0e-042	4.6e-048

Enriched Matching Words

Word	Positives	Negatives	P-value	E-value
CTCATAA	92 / 19952	13 / 19952	3.7e-016	6.8e-012
CTCTTAA	85 / 19952	12 / 19952	4.9e-015	9.0e-011
CTCCTAA	96 / 19952	21 / 19952	5.6e-013	1.0e-008
CTCGTAA	37 / 19952	6 / 19952	8.1e-007	1.5e-002

	Motif	Logo	RC Logo	E-value	Unerased E-value	More	Submit/Dov
9.	AGGTCSY	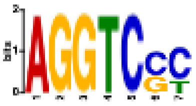	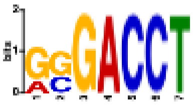	6.0e-037	1.0e-035	↥	⇢

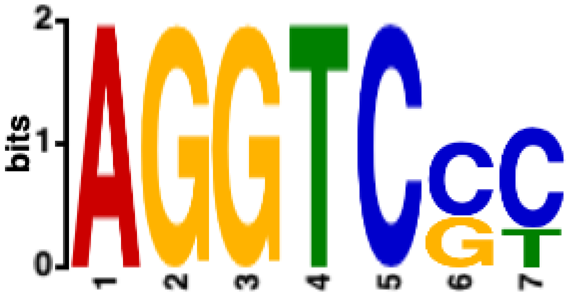

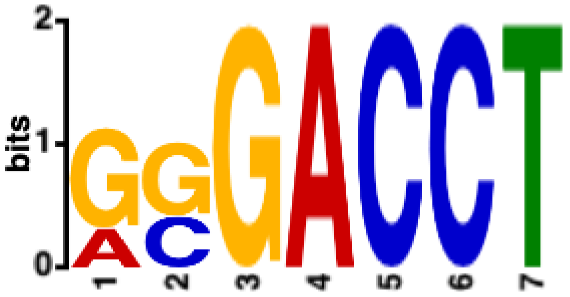

Details

Positives	Negatives	P-value	E-value	Unerased E-value
358 / 19952	86 / 19952	3.3e-041	6.0e-037	1.0e-035

Enriched Matching Words

Word	Positives	Negatives	P-value	E-value
AGGTCCC	148 / 19952	31 / 19952	7.4e-020	1.3e-015
AGGTCGC	110 / 19952	23 / 19952	3.8e-015	6.9e-011
AGGTCCT	64 / 19952	15 / 19952	1.1e-008	2.0e-004
AGGTCGT	36 / 19952	17 / 19952	6.3e-003	1.1e+002

Motif	Logo	RC Logo	E-value	Unerased E-value	More	Submit/Dov

	Motif	Logo	RC Logo	E-value	Unerased E-value	More	Submit/Dov
**10.**	AYTTTTR	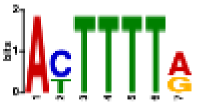	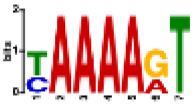	8.0e-031	2.2e-052		

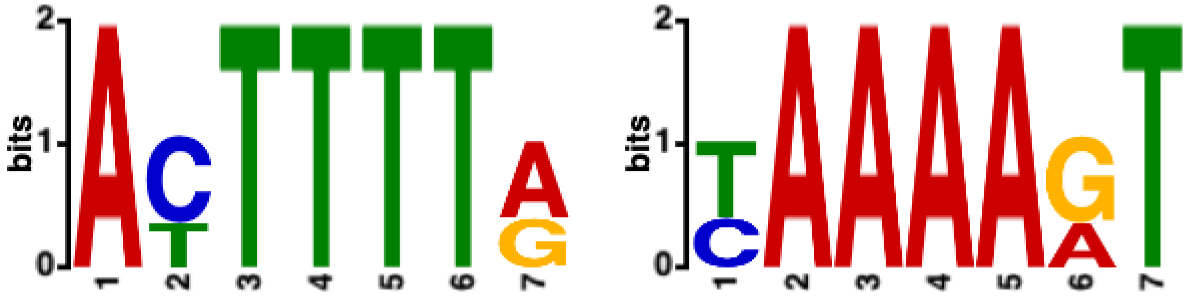

Details

Positives	Negatives	P-value	E-value	Unerased E-value
317 / 19952	80 / 19952	4.4e-035	8.0e-031	2.2e-052

Enriched Matching Words

Word	Positives	Negatives	P-value	E-value
ACTTTTA	143 / 19952	20 / 19952	1.8e-024	3.2e-020
ACTTTTG	75 / 19952	24 / 19952	1.3e-007	2.4e-003
ATTTTTA	58 / 19952	18 / 19952	2.3e-006	4.2e-002
ATTTTTG	49 / 19952	18 / 19952	9.6e-005	1.7e+000

	Motif	Logo	RC Logo	E-value	Unerased E-value	More	Submit/Dov
**11.**	AATGGWTA	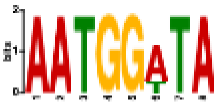	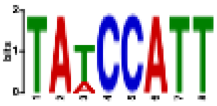	7.2e-025	7.2e-025		
**12.**	TKYAAA	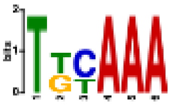	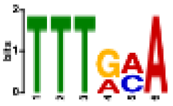	1.0e-026	1.1e-066		
**13.**	AAAACD	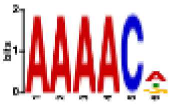	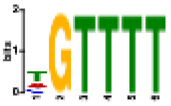	1.0e-021	7.4e-023		
**14.**	CCACHGAG	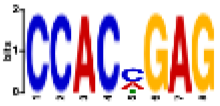	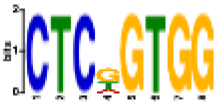	2.0e-020	1.6e-023		
**15.**	TCGAAGGR	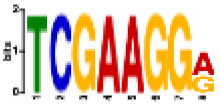	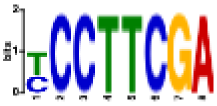	2.2e-019	2.2e-019		
**16.**	ACTWCGGA	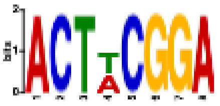	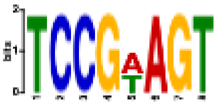	4.3e-019	4.3e-019		
**17.**	TAAGSCR	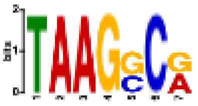	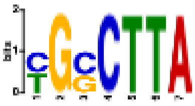	4.5e-030	2.5e-046		
**18.**	ACRTGAT	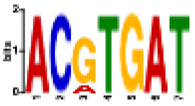	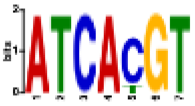	3.6e-018	9.8e-039		

	Motif	Logo	RC Logo	E-value	Unerased E-value	More	Submit/Dov
19.	GGWTTCGA	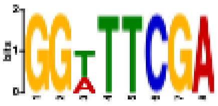	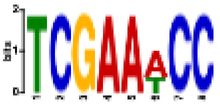	4.0e-018	1.5e-074		
20.	CGCCCRA	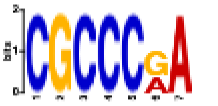	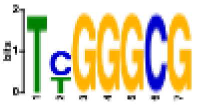	6.3e-018	4.2e-017		
21.	AYCAGAA	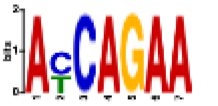	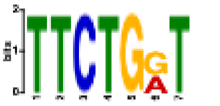	6.8e-017	3.6e-023		
22.	AGGSTCCA	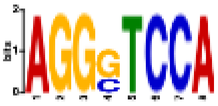	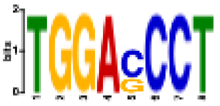	1.6e-016	1.6e-016		
23.	CATCAGMC	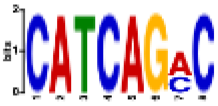	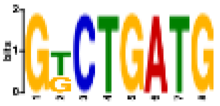	1.6e-016	5.0e-022		
24.	GAGCMGGA	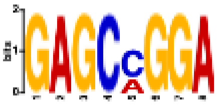	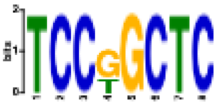	1.4e-015	1.2e-016		
25.	ACCNACTG	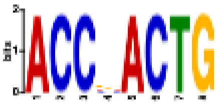	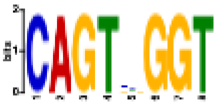	2.5e-015	5.0e-031		
26.	ACCVAAAG	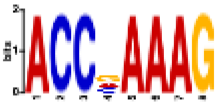	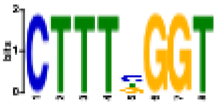	3.0e-015	1.9e-015		
27.	ATCCTWA	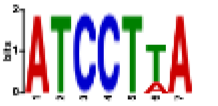	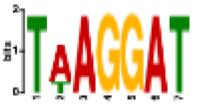	3.6e-015	2.2e-017		
28.	ATWGTG	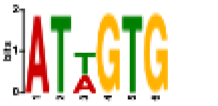	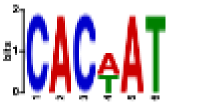	2.6e-014	2.6e-014		
29.	CGGTGARA	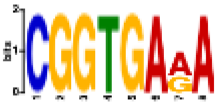	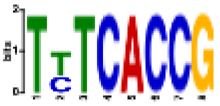	9.6e-014	9.6e-014		
30.	ACGGWAGT	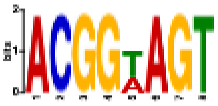	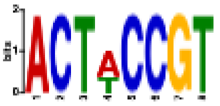	1.1e-013	3.0e-016		
31.	TGGATTYA	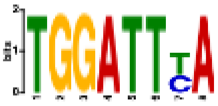	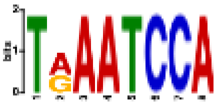	1.5e-013	1.6e-015		
32.	CAGGGHCG	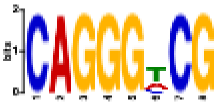	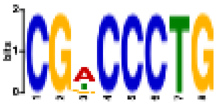	1.6e-013	6.2e-013		
33.	ACAGGCYA	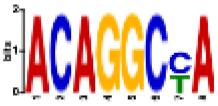	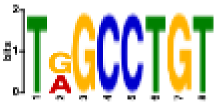	2.1e-013	2.1e-013		
34.	GCTGSGGA	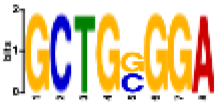	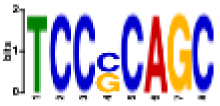	3.5e-012	4.8e-013		
35.	CCCAVACC	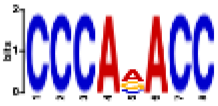	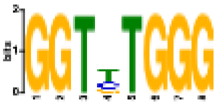	3.5e-012	1.4e-011		

	Motif	Logo	RC Logo	E-value	Unerased E-value	More	Submit/Dov
**36.**	GACATGVC	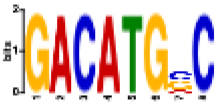	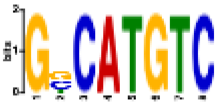	1.0e-011	1.0e-011	↧	⇢
**37.**	AGCGCRT	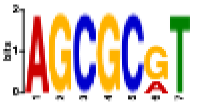	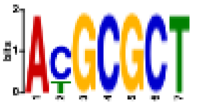	2.4e-011	3.4e-015	↧	⇢
**38.**	AYAGCCG	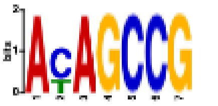	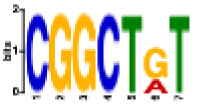	3.3e-011	3.6e-027	↧	⇢
**39.**	ATTAGCAS	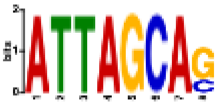	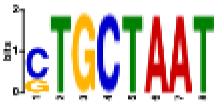	9.7e-011	9.7e-011	↧	⇢
**40.**	AACCTGY	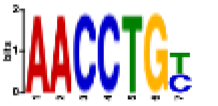	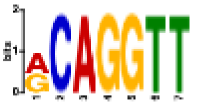	1.1e-010	2.2e-023	↧	⇢
**41.**	AGTGGWTA	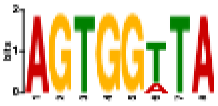	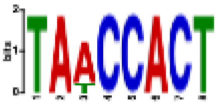	6.9e-010	7.8e-014	↧	⇢
**42.**	CTTAMGTC	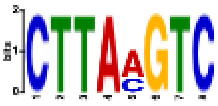	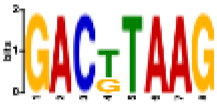	7.3e-010	8.4e-008	↧	⇢
**43.**	GTGCCTGA	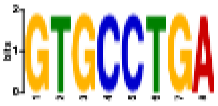	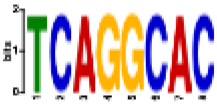	9.2e-010	9.2e-010	↧	⇢
**44.**	ACYAACGA	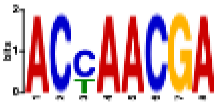	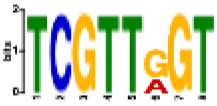	9.5e-010	9.5e-010	↧	⇢
**45.**	CAGATGBG	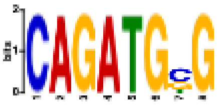	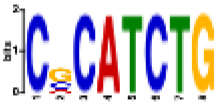	1.8e-009	7.3e-009	↧	⇢
**46.**	CTCSCGTA	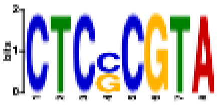	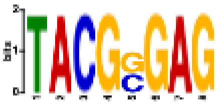	5.5e-009	5.5e-009	↧	⇢
**47.**	AGAAGYCC	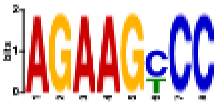	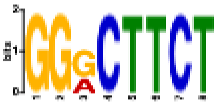	1.0e-008	3.3e-009	↧	⇢
**48.**	GCCSGGM	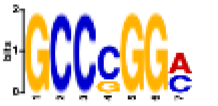	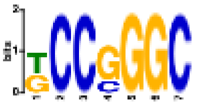	8.2e-009	3.7e-012	↧	⇢
**49.**	CTTTTCCB	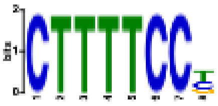	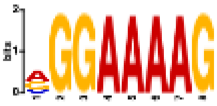	2.3e-008	2.3e-008	↧	⇢
**50.**	GAYAGGTA	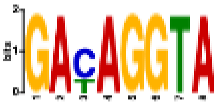	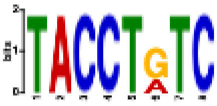	4.4e-008	4.4e-008	↧	⇢
**51.**	AMGTGGGG	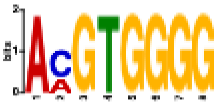	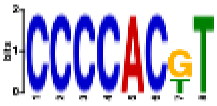	4.7e-008	1.4e-006	↧	⇢
**52.**	CTCCGGWG	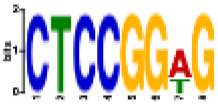	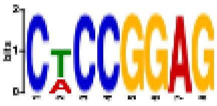	5.3e-008	5.3e-008	↧	⇢

	Motif	Logo	RC Logo	E-value	Unerased E-value	More	Submit/Dov
**53.**	CBACGAGG	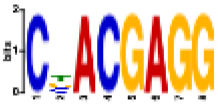	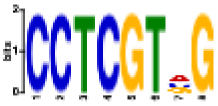	6.0e-008	6.0e-008	↥	⇢

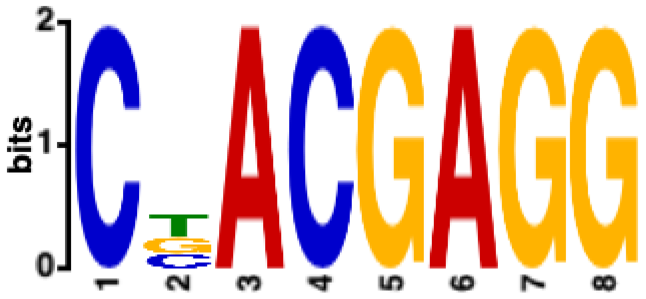

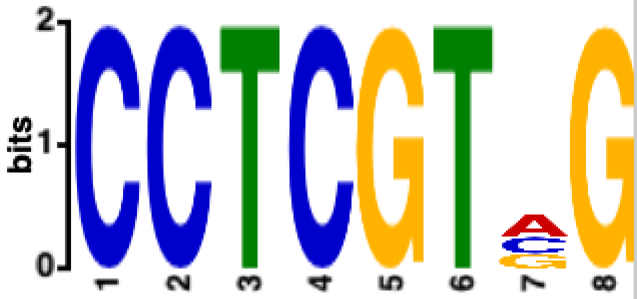

Details

Positives	Negatives	P-value	E-value	Unerased E-value
66 / 19952	9 / 19952	3.7e-012	6.0e-008	6.0e-008

Enriched Matching Words

Word	Positives	Negatives	P-value	E-value
CTACGAGG	28 / 19952	2 / 19952	4.3e-007	7.0e-003
CGACGAGG	20 / 19952	3 / 19952	2.4e-004	4.0e+000
CCACGAGG	18 / 19952	4 / 19952	2.2e-003	3.5e+001

	Motif	Logo	RC Logo	E-value	Unerased E-value	More	Submit/Dov
**54.**	AMTGTCAG	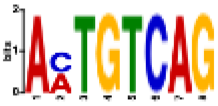	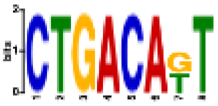	8.2e-008	4.3e-007	↧	⇢
**55.**	AGAGMGCC	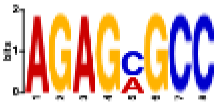	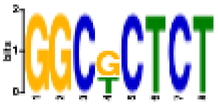	1.0e-007	1.8e-006	↧	⇢
**56.**	ACACCTCM	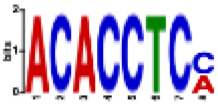	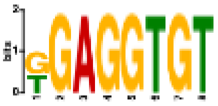	1.5e-007	1.5e-007	↧	⇢
**57.**	CAGTCWAA	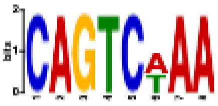	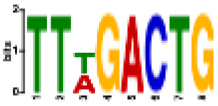	1.1e-007	1.1e-007	↧	⇢
**58.**	ATCCAWTG	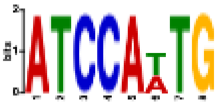	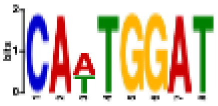	1.5e-007	3.5e-013	↧	⇢
**59.**	GAGTCGW	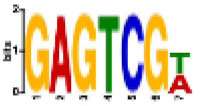	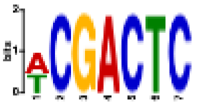	1.5e-007	1.7e-017	↧	⇢
**60.**	AGAGMAAT	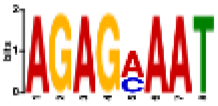	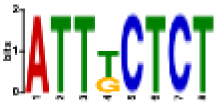	1.6e-007	1.1e-008	↧	⇢
**61.**	KGCTATAA	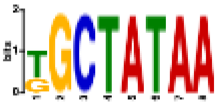	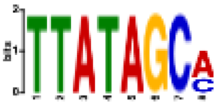	1.6e-007	1.6e-007	↧	⇢
**62.**	AAGCGTCC	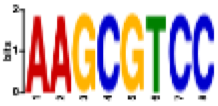	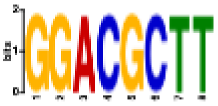	2.3e-007	1.1e-008	↧	⇢

	Motif	Logo	RC Logo	E-value	Unerased E-value	More	Submit/Dov
**63.**	CCASTGGC	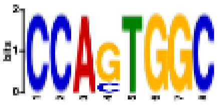	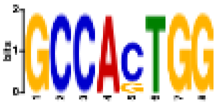	2.7e-007	3.5e-009	↧	⇢
**64.**	CCAAYGC	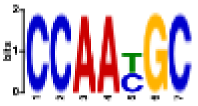	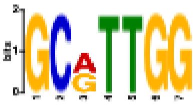	2.9e-007	1.2e-015	↧	⇢
**65.**	ATTTTRT	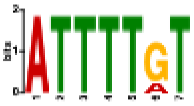	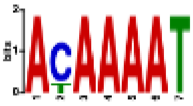	4.1e-007	1.7e-005	↧	⇢
**66.**	ATATGKCC	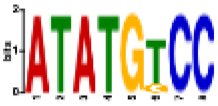	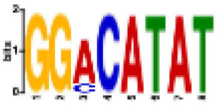	4.6e-007	4.6e-007	↧	⇢
**67.**	CAGGGAM	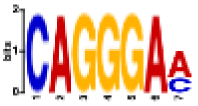	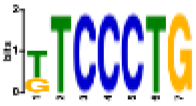	4.4e-007	1.4e-008	↧	⇢
**68.**	ACRCAGCC	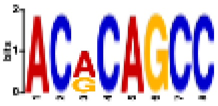	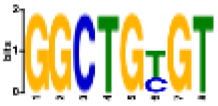	5.0e-007	1.1e-005	↧	⇢
**69.**	CCCATAAC	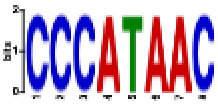	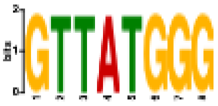	9.1e-007	9.1e-007	↧	⇢
**70.**	ACCCCTAG	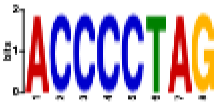	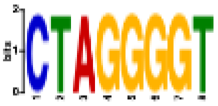	1.8e-006	1.8e-006	↧	⇢
**71.**	CCCCTCTK	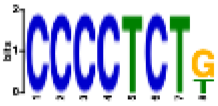	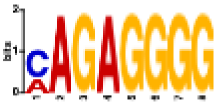	1.8e-006	1.8e-006	↧	⇢
**72.**	ATTGCGCC	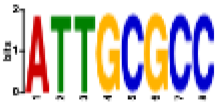	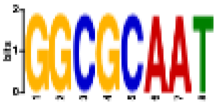	1.9e-006	6.9e-009	↧	⇢
**73.**	GACACSGA	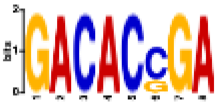	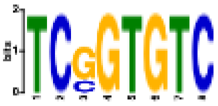	1.9e-006	1.9e-006	↧	⇢
**74.**	CTGGGTRG	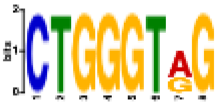	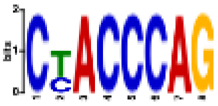	2.1e-006	6.8e-007	↧	⇢
**75.**	CGAAGGAR	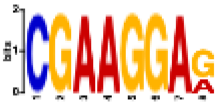	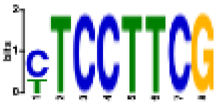	8.4e-006	3.3e-012	↧	⇢
**76.**	AACAGSAG	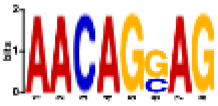	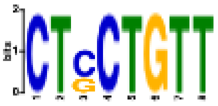	9.9e-006	3.3e-007	↧	⇢
**77.**	GGTAAWGA	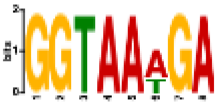	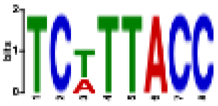	1.2e-005	1.2e-005	↧	⇢
**78.**	CTAGAYAG	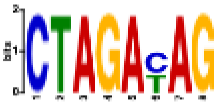	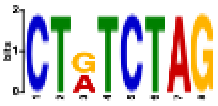	3.1e-005	3.5e-006	↧	⇢
**79.**	ACTGKAG	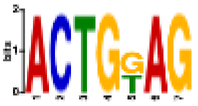	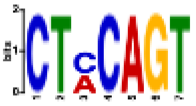	3.1e-005	4.5e-010	↧	⇢

	Motif	Logo	RC Logo	E-value	Unerased E-value	More	Submit/Dov
**80.**	CTCGCTTH	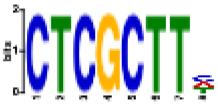	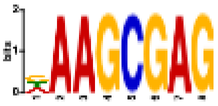	3.2e-005	2.9e-009		
**81.**	CGCGKCAG	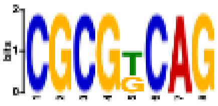	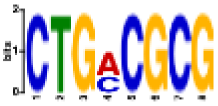	3.3e-005	2.5e-007		
**82.**	ACCGGTAG	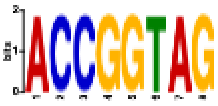	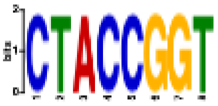	6.1e-005	6.1e-005		
**83.**	ACGGAAAC	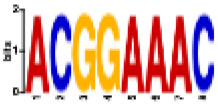	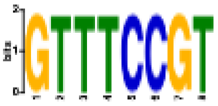	7.9e-005	8.7e-010		
**84.**	CWAAAACC	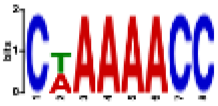	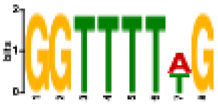	9.0e-005	8.9e-008		
**85.**	AGCCWGCA	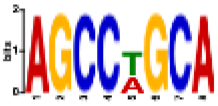	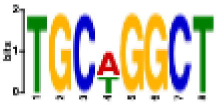	1.3e-004	2.3e-008		
**86.**	AACCMTGG	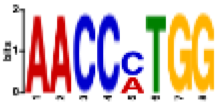	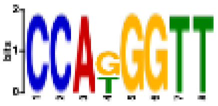	2.5e-004	2.1e-008		
**87.**	GAAAATGA	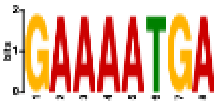	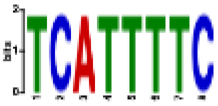	4.4e-004	4.4e-004		
**88.**	GCATGC	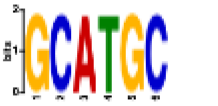	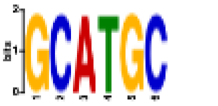	4.6e-004	3.1e-005		
**89.**	CGKGTACG	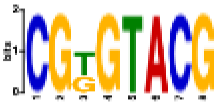	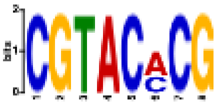	5.3e-004	5.3e-004		
**90.**	AGRGGATG	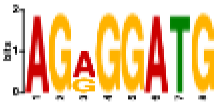	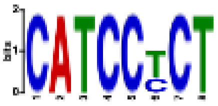	5.3e-004	5.3e-004		
**91.**	AWCGGTTA	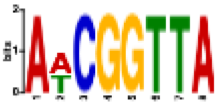	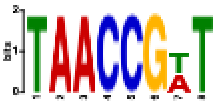	6.8e-004	6.8e-004		
**92.**	AGGAAART	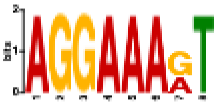	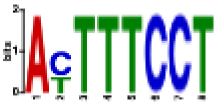	7.6e-004	5.5e-003		
**93.**	TCTCKACA	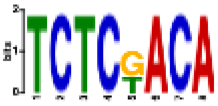	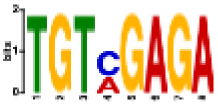	7.8e-004	2.9e-007		
**94.**	ACAGATAM	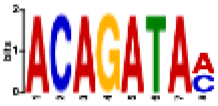	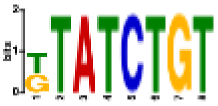	8.5e-004	8.5e-004		
**95.**	MCACGCCA	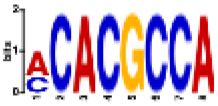	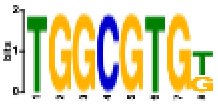	1.0e-003	5.8e-003		
**96.**	CGATGGAY	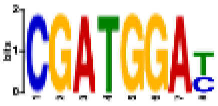	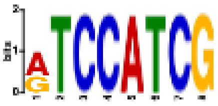	1.3e-003	1.3e-003		

	Motif	Logo	RC Logo	E-value	Unerased E-value	More	Submit/Dov
97.	CCTTTAGA	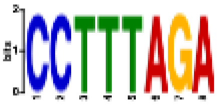	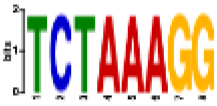	1.8e-003	1.6e-003		
98.	CSTGAAGA	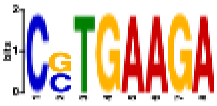	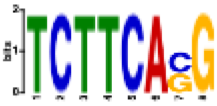	1.3e-003	4.7e-003		
99.	CAGTTMCC	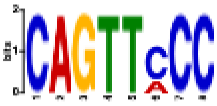	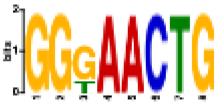	1.9e-003	1.8e-005		
100.	ACTTCSTC	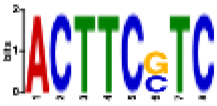	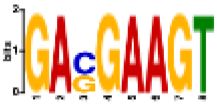	2.5e-003	3.3e-004		
101.	GGTCTCMC	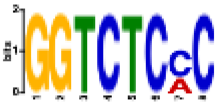	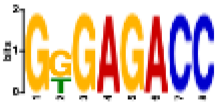	2.7e-003	1.6e-003		
102.	TACSGGGA	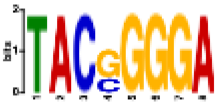	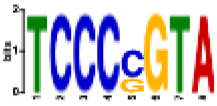	3.2e-003	9.3e-004		
103.	AAATAACC	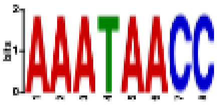	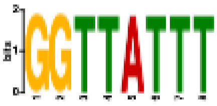	3.6e-003	3.6e-003		
104.	CCCMCTTC	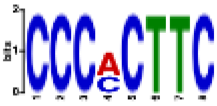	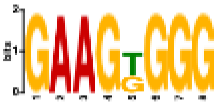	4.6e-003	1.3e-004		
105.	TCGTGGCR	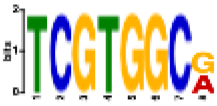	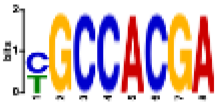	4.6e-003	4.6e-003		
106.	RCGAGCCA	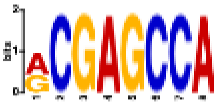	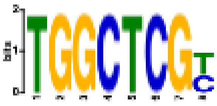	5.2e-003	2.0e-004		
107.	ACARGTGG	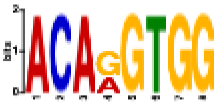	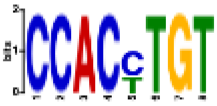	5.8e-003	5.8e-003		
108.	AATTTTAG	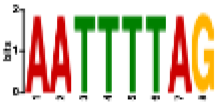	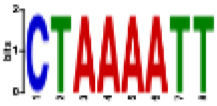	8.2e-003	2.3e-004		
109.	ACCACCAA	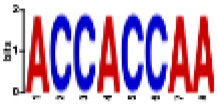	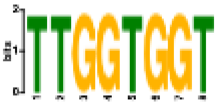	8.1e-003	4.4e-008		
110.	CACATTCG	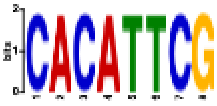	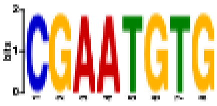	8.1e-003	4.3e-011		
111.	ACAMGCAG	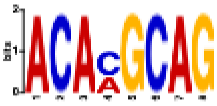	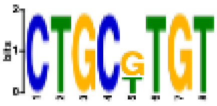	1.0e-002	6.7e-012		
112.	CRACTTGC	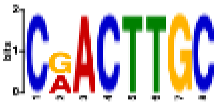	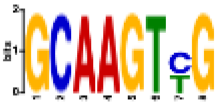	1.0e-002	1.0e-002		
113.	CCMGATGA	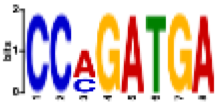	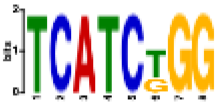	1.2e-002	1.6e-005		

	Motif	Logo	RC Logo	E-value	Unerased E-value	More	Submit/Dov
114.	ACCTAGWA	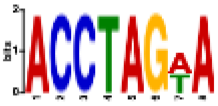	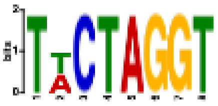	1.2e-002	1.8e-003	↧	⇢
115.	AGRATCAT	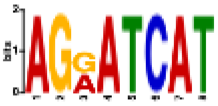	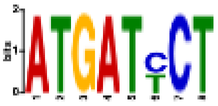	1.2e-002	1.2e-002	↧	⇢
116.	CCACTAGA	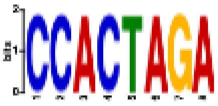	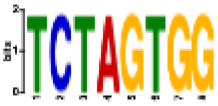	1.2e-002	3.5e-003	↧	⇢
117.	GGAGACMA	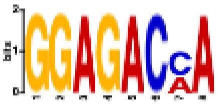	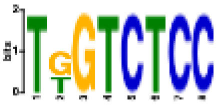	1.2e-002	2.0e-003	↧	⇢
118.	CTCGGRTC	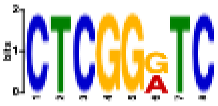	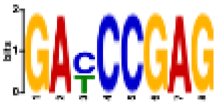	2.1e-002	2.3e-003	↧	⇢
119.	GGATTWGA	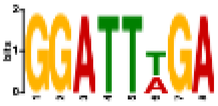	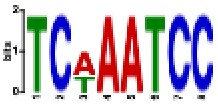	2.3e-002	3.4e-038	↧	⇢
120.	GGSTCACC	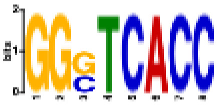	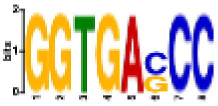	2.6e-002	1.4e-003	↧	⇢
121.	GRGATTAA	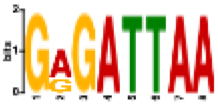	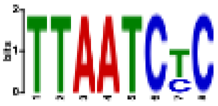	2.6e-002	2.2e-011	↧	⇢
122.	GTTAAGTM	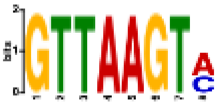	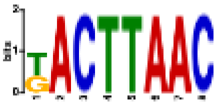	2.8e-002	2.8e-002	↧	⇢
123.	ACCTCCMG	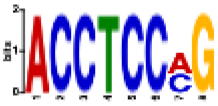	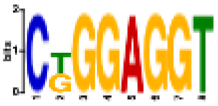	3.2e-002	3.1e-001	↧	⇢
124.	ACACAWAT	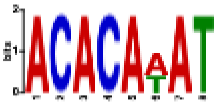	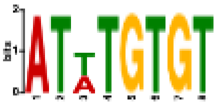	3.5e-002	1.9e-002	↧	⇢
125.	ARAGAGGC	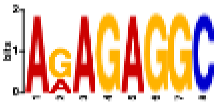	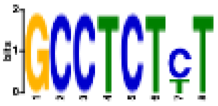	4.1e-002	4.1e-002	↧	⇢
126.	AACCGWAG	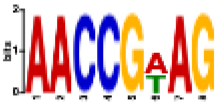	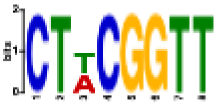	4.1e-002	4.1e-002	↧	⇢
127.	AACCSGGG	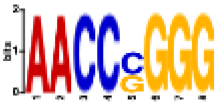	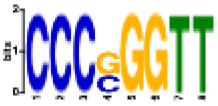	4.1e-002	2.5e-029	↧	⇢
128.	AAGRTTGC	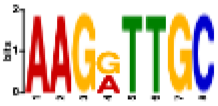	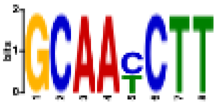	4.2e-002	1.4e-004	↧	⇢
129.	GAGCAAAA	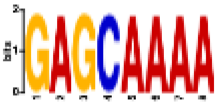	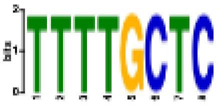	4.2e-002	9.7e-014	↧	⇢
130.	TGTCMACA	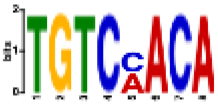	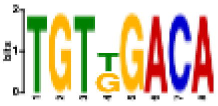	4.2e-002	2.2e-002	↧	⇢

Inputs & Settings

Previous Next Top

Sequences

Source	Alphabet	Sequence Count
./seqs-centered	DNA	19952

Control Sequences

Source	Sequence Count
./seqs-shuffled	19952

Background

Name	Bg.				Bg.	Name
Adenine	0.187	A	~	T	0.294	Thymine
Cytosine	0.249	C	~	G	0.270	Guanine

Other Settings

**Strand Handling**	Both the given and reverse complement strands are processed
**# REs to Generalize**	100
**Shuffle Seed**	1
**E-value Threshold**	0.05
**Max Motif Count**	No maximum motif count.
**Max Run Time**	7099 seconds.

Previous Top

DREME version
5.0.5 (Release date: Mon Mar 18 20:12:19 2019 -0700)

Reference
Timothy L. Bailey, "DREME: Motif discovery in transcription factor ChIP-seq data", *Bioinformatics*, 27(12):1653-1659, 2011. [full text]

Command line

```
dreme -verbosity 1 -oc dreme_out -png -dna -p ./seqs-centered -n ./seqs-shuffled -t 7099 -e 0.05
```
